# Analysis of Cutting Stability of a Composite Variable-Section Boring Bar with a Large Length-to-Diameter Ratio Considering Internal Damping

**DOI:** 10.3390/ma15155465

**Published:** 2022-08-08

**Authors:** Jingmin Ma, Jianfeng Xu, Longfei Li, Xingguang Liu, Ming Gao

**Affiliations:** 1 College of Energy and Mining Engineering, Shandong University of Science and Technology, Qingdao 266590, China; 2 Mechanical and Electronic Engineering College, Shandong Provincial Key Laboratory of Horticultural Machineries and Equipments, Shandong Agricultural Equipment Intelligent Engineering Laboratory, Shandong Agricultural University, Tai’an 271018, China

**Keywords:** Euler–Bernoulli beam theory, composite variable-section boring bar, cutting stability, large length-to-diameter ratio, time-domain method

## Abstract

Chattering in composite deep-hole boring can directly affect surface processing quality and efficiency and has always been a research hotspot in machining mechanics. In this study, based on Euler–Bernoulli beam theory, the fine control equations for the cutting stability of composite variable-section boring bars were established using the Hamilton principle, in which the sectional change and internal damping of the material were considered. Next, using the Galerkin method and semi-discrete method, the effects of the taper ratio, damping ratio, length-to-diameter ratio, and ply angle on the free vibration characteristics and cutting stability were analyzed in detail. The results show that at a low damping ratio, both the first-order inherent frequency and boring stability can be enhanced with the increase in the taper ratio; at a large damping ratio, increasing the taper ratio can reduce the first-order inherent frequency and boring stability. Finally, the effects of the sectional change on the inherent frequency, displacement response, and convergence were analyzed. A numerical simulation was performed for the model reliability validation. The present research results can provide a theoretical basis and technical guidance for analyzing the cutting stability and fine control of composite variable-section boring bars with large length-to-diameter ratios.

## 1. Introduction

Some deep holes, such as cylinder holes, axial oil holes of the shaft, rocket projectiles, and barrels of various artilleries, require high machining precision and surface quality, whereas some materials to be processed show poor cutting machinability. These have become a great challenge in production. Meanwhile, the boring bar of deep-hole processing is restricted by the hole diameter, showing a great length-to-diameter ratio, poor first-order bending rigidity, and low strength. Under the vortex motion of the boring bar, some problems including vibration, ripples, and tapers are easily generated, which can affect the linearity and surface roughness of deep holes, thereby inducing a series of processing problems such as noise [1]. A good-quality boring bar with a large length-to-diameter ratio should have high static rigidity, high dynamic rigidity, and high first-order natural bending frequency so as to avoid the chattering and vortex motion generated in high-speed rotation [2,3]. The suitable selection of the cutting parameters and size of the boring bar to enhance stability in cutting are key to enhancing the processing efficiency and quality in deep-hole boring as well as deep-hole processing capabilities. Chatter prediction and control in deep-hole boring has become a hot topic in machining dynamics.

The stability lobe diagram, proposed by Merritt in 1965 [4], is now the most commonly used method for cutting stability analysis. The stability lobe diagram can describe the relationship between the rotation speed of the cutting main shaft and the cutting dosage. During machining technological design, the cutting dosage can be appropriately selected in accordance with the lobe diagram to effectively inhibit chattering. Currently, scholars mainly use the time-domain method [5], frequency method [6], and analysis-experiment method [7] to plot the stability lobe diagram. Some scholars have performed a full discretization on the time-delay differential equation using the time-domain full discretization method and calculated the transition matrix via numerical iteration to reduce the complexity of the discrete iterative formula [8]. Owing to the advantages of high intensity, large rigidity, lightweight, anti-fatigue, shock absorption, high-temperature resistance, and favorable designability, a composite boring bar with a large length-to-diameter ratio can more remarkably enhance the rigidity and first-order natural bending frequency than a traditional metal boring bar. However, in contrast with ordinary metal materials, composite materials possess larger internal damping [9], which increases the complexity of analyzing the inherent vibration frequency [10]. Due to the existence of damping, the rotor can generate an unstable vibration region, which is contrary to the hindrance of damping on rotation in conventional cognition [11,12]. This unstable region can directly affect the enhancement of the rotation speed and cutting efficiency. Scholars [13,14,15] have conducted a great deal of research into the stability of metal boring bars using calculations and nonlinear analysis. Kim et al. [16] comprehensively considered the external viscous damping, internal viscous damping, and the taper of a boring bar and analyzed the free vibration characteristics of a composite variable-section boring bar and its stability in boring and milling. Their experimental results not only theoretically proved the enhancement of system stiffness and cutting stability by the taper, but also confirmed the different effects of internal damping on the cutting stability during low-speed and high-speed rotations. However, this model neglects the effects of the inertial force in the vibration equation. Since a boring bar rotates at high speeds during the boring process, it is necessary to consider the gyroscopic effect on system stability [17]. Ma et al. [18] analyzed the cutting stability of composite boring bars and investigated the influences of internal and external damping, ply orientation, gyroscopic effect, and the taper ratio of boring bars while ignoring the effects of the cross-section variations in a variable-section boring bar.

In order to address the existing problems, this study innovatively established the fine cutting stability analysis model for composite variable-section boring bars by taking into account factors including inner damping, gyroscopic effect, sectional change, and taper ratio. The influencing rules of material damping, ply angle, sectional change, and length-to-diameter ratio on the free vibration characteristics and cutting stability of the boring bar were analyzed. According to the numerical analysis results, it can be concluded that the sectional change imposed a significant effect on the calculation results. Furthermore, this study innovatively analyzed the cutting stability of the boring bar by simultaneously considering the sectional change and the damping ratio.

## 2. Free Vibration Control Equation and Solution to Vibration Characteristics

**Figure 1:**
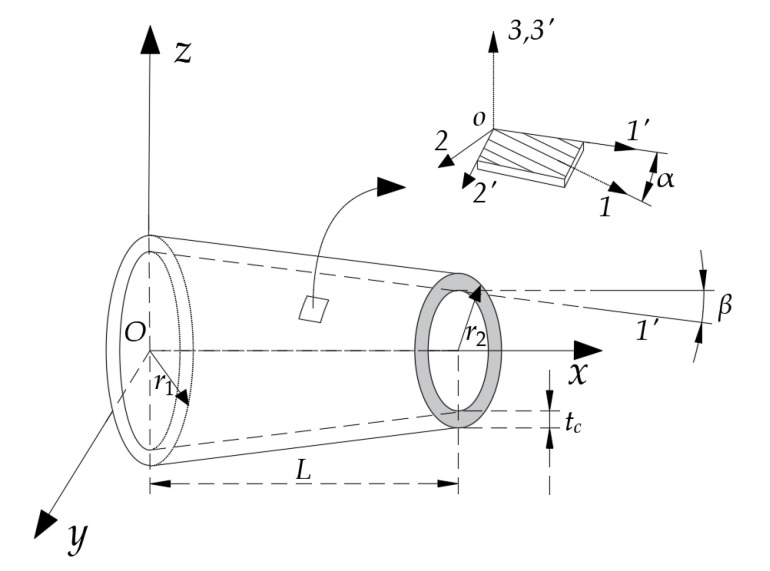
Illustration of the simplified boring bar model.

The boring bar in this study was simplified into a variable-section hollow slender rod model, as shown in Figure 1. In this figure, *O-xyz* denotes the global fixed coordinate system in which the x-axis points toward the axial direction of the boring bar, and the *y*-axis and *z*-axis are located on the cross-section of the boring bar and point along the radius direction; *o-123* and *o-1′2′3′* are two local coordinate systems that reflect the fiber ply angle, in which the *1*-axis points along the fiber direction, the *1′*-axis points toward the axis direction of the boring bar, the *2*-axis is located in the plane where the fibers are located and is perpendicular to the fiber direction, the *3*-axis is perpendicular to the direction of the plane where the fibers are located; α denotes the intersection angle between the main ply direction *1* of the fibers and axis, β denotes the taper angle of the variable-section rod, $r_{1}$, $r_{2}$, $t_{c}$, and *L* are the radius of the root section, the radius of the end section, and the thickness and length of the boring bar, respectively.

### 2.1. Constitutive Equation of the Boring Bar after the Consideration of Material Damping

The composite boring bar in this study is anisotropic and the constitutive equation can be expressed as

(1) $$\left\{\begin{array}{c} \sigma_{1}\\ \sigma_{2}\\ \sigma_{3}\\ \tau_{23}\\ \tau_{31}\\ \tau_{12} \end{array}\right\}=\left[\begin{array}{cccccc} Q_{11} & Q_{12} & Q_{13} & 0 & 0 & 0\\ Q_{12} & Q_{22} & Q_{23} & 0 & 0 & 0\\ Q_{13} & Q_{23} & Q_{33} & 0 & 0 & 0\\ 0 & 0 & 0 & Q_{44} & 0 & 0\\ 0 & 0 & 0 & 0 & Q_{55} & 0\\ 0 & 0 & 0 & 0 & 0 & Q_{66} \end{array}\right]\left\{\begin{array}{c} \varepsilon_{1}\\ \varepsilon_{2}\\ \varepsilon_{3}\\ \gamma_{23}\\ \gamma_{31}\\ \gamma_{12} \end{array}\right\}$$

where

(2) $$\begin{array}{c} Q_{11}=\frac{1-v_{23}v_{32}}{E_{2}E_{3}\Delta },Q_{12}=\frac{v_{21}+v_{31}v_{23}}{E_{2}E_{3}\Delta }=\frac{v_{12}+v_{32}v_{13}}{E_{1}E_{3}\Delta },Q_{13}=\frac{v_{31}+v_{21}v_{32}}{E_{2}E_{3}\Delta }=\frac{v_{13}+v_{12}v_{23}}{E_{1}E_{2}\Delta },\\ Q_{22}=\frac{1-v_{13}v_{31}}{E_{1}E_{3}\Delta },Q_{23}=\frac{v_{32}+v_{12}v_{31}}{E_{1}E_{3}\Delta }=\frac{v_{23}+v_{21}v_{13}}{E_{1}E_{2}\Delta },Q_{33}=\frac{1-v_{12}v_{21}}{E_{1}E_{2}\Delta },\\ Q_{44}=G_{23},Q_{55}=G_{31},Q_{66}=G_{12},\\ \Delta =\frac{1-v_{12}v_{21}-v_{23}v_{32}-v_{13}v_{31}-2v_{21}v_{32}v_{13}}{E_{1}E_{2}E_{3}} \end{array}$$

Given the viscoelastic properties of the composite materials, the stress can be divided into elastic stress $\left\{\sigma_{e}\right\}$ and dissipative stress $\left\{\sigma_{d}\right\}$:

(3) $$\left\{\sigma \right\}=\left\{\sigma_{e}\right\}+\left\{\sigma_{d}\right\}$$

which can further be expressed as

(4) $$\left\{\sigma \right\}=\left[Q\right]\left\{\varepsilon \right\}+{\left[Q\right]}^{\psi }\left\{\dot{\varepsilon }\right\}$$

where ${\left[Q\right]}^{\psi }=\left[Q\right]\left[\eta \right]$ denotes the damping stiffness matrix of the material. [η] denotes the damping matrix of various layers and is related to their dissipation characteristics, which can be expressed as

(5) $$\left[\eta \right]=\frac{\pi }{2}\left[\psi \right]$$

The dissipation characteristics of the various layers can be described by the following damping ratio matrix

(6) $$\left[\psi \right]=\left[\begin{array}{cccccc} \psi_{1} & 0 & 0 & 0 & 0 & 0\\ 0 & \psi_{2} & 0 & 0 & 0 & 0\\ 0 & 0 & \psi_{3} & 0 & 0 & 0\\ 0 & 0 & 0 & \psi_{23} & 0 & 0\\ 0 & 0 & 0 & 0 & \psi_{13} & 0\\ 0 & 0 & 0 & 0 & 0 & \psi_{12} \end{array}\right]$$

Therefore, the damping stiffness matrix ${\left[Q\right]}^{\psi }$ can be described by the damping ratio matrix [ψ] as

(7) $${\left[Q\right]}^{\psi }=\frac{\pi }{2}\left[Q\right]\left[\psi \right]$$

Assume that α denotes the intersection angle between any fiber ply in the laminated plate and the axial *x*-axis direction, as shown in Figure 1. The relationship between stress and strain in the global coordinate system can be expressed as

(8) $$\left\{\sigma \right\}=\left[\overset{–}{Q}\right]\left\{\varepsilon \right\}+{\left[\overset{–}{Q}\right]}^{\psi }\left\{\dot{\varepsilon }\right\}$$

where $\left[\overset{–}{Q}\right]$ and ${\left[\overset{–}{Q}\right]}^{\psi }$ denote the stiffness matrix and the damping stiffness matrix in the global coordinate system that have been converted by the matrices in the local coordinate system, respectively, which can be expressed as

(9a) $$\left[\overset{–}{Q}\right]={\left[T_{\beta }\right]}^{-1}{\left[T_{\alpha }\right]}^{-1}\left[Q\right]\left[T_{\alpha }\right][T_{\beta }]$$

(9b) $${\left[\overset{–}{Q}\right]}^{\psi }={\left[T_{\beta }\right]}^{-1}{\left[T_{\alpha }\right]}^{-1}{\left[Q\right]}^{\psi }\left[T_{\alpha }\right][T_{\beta }]$$

The matrices for the coordinate transformation can be written as

(10a) $$\left[T_{\alpha }\right]=\left[\begin{array}{cccccc} \cos^{2}\alpha & \sin^{2}\alpha & 0 & 0 & 0 & \cos\alpha \sin\alpha \\ \sin^{2}\alpha & \cos^{2}\alpha & 0 & 0 & 0 & -\cos\alpha \sin\alpha \\ 0 & 0 & 1 & 0 & 0 & 0\\ 0 & 0 & 0 & \cos\alpha & -\sin\alpha & 0\\ 0 & 0 & 0 & \sin\alpha & \cos\alpha & 0\\ -2\cos\alpha \sin\alpha & 2\cos\alpha \sin\alpha & 0 & 0 & 0 & \cos^{2}\alpha -\sin^{2}\alpha \end{array}\right]$$

(10b) $$\left[T_{\beta }\right]=\left[\begin{array}{cccccc} \sin^{2}\beta & 0 & \cos^{2}\beta & 0 & -\sin\beta \cos\beta & 0\\ 0 & 1 & 0 & 0 & 0 & 0\\ \cos^{2}\beta & 0 & \sin^{2}\beta & 0 & \sin\beta \cos\beta & 0\\ 0 & 0 & 0 & -\sin\beta & 0 & -\cos\beta \\ 2\sin\beta \cos\beta & 0 & -2\sin\beta \cos\beta & 0 & \sin^{2}\beta -\cos^{2}\beta & 0\\ 0 & 0 & 0 & \cos\beta & 0 & -\sin\beta \end{array}\right]$$

Considering the plane stress state, the elastic stress and dissipative stress can be expressed as

(11a) $${\left\{\sigma \right\}}_{e}=\{\begin{array}{l} \sigma_{xx}{}^{e}={\overset{–}{K}}_{11}\varepsilon_{xx}\\ \tau_{xz}{}^{e}=k_{s}{\overset{–}{K}}_{12}\gamma_{xz}\\ \tau_{xy}{}^{e}=k_{s}{\overset{–}{K}}_{13}\gamma_{xy} \end{array}$$

(11b) $${\left\{\sigma \right\}}_{d}=\{\begin{array}{l} \sigma_{xx}{}^{d}={\overset{–}{K}}_{11}^{\psi }{\dot{\varepsilon }}_{xx}\\ \tau_{xz}{}^{d}=k_{s}{\overset{–}{K}}_{12}^{\psi }{\dot{\gamma }}_{xz}\\ \tau_{xy}{}^{d}=k_{s}{\overset{–}{K}}_{13}^{\psi }{\dot{\gamma }}_{xy} \end{array}$$

where $\left[K_{ij}\right]$ and ${\left[K_{ij}\right]}^{\psi }$ are shown in Appendix A and $k_{s}$ denotes the shear correction factor.

### 2.2. Elastic Strain Energy of the Boring Bar

Under the plane stress state, the elastic strain energy of the boring bar can be written as

(12) $$U=\frac{1}{2}\int_{0}^{L}\int_{S}\left(\sigma_{xx}{}^{e}\varepsilon_{xx}+\tau_{xz}{}^{e}\gamma_{xz}+\tau_{xy}{}^{e}\gamma_{xy}\right)dSdx$$

Ma et al. confirmed that when the length-to-diameter ratio (*L*/2*R*) exceeded 5, the Euler–Bernoulli beam theory showed high precision for anisotropic steel bars [19]; for the anisotropic composite bar, the value of *L*/2*R* should be greater than 8 to achieve the error of below 5%. Since the length-to-diameter ratio of the boring bar in this study exceeded 8, the Euler–Bernoulli beam theory can satisfy the actual requirements in engineering. Based on the Euler–Bernoulli beam theory, the shear stress induced by the bending and torsion on the cross-section can be ignored, i.e., $\tau_{xz}^{e}=\tau_{xy}^{e}=0$. Simultaneously, considering small deformations, the axial strain induced by bending can be expressed as

(13) $$\varepsilon_{xx}=z\frac{\partial \theta_{y}}{\partial x}-y\frac{\partial \theta_{z}}{\partial x}$$

(14) $$\theta_{y}=-\frac{\partial w}{\partial x},\;\theta_{z}=\frac{\partial v}{\partial x}$$

where *v* and *w* denote the lateral displacements of the boring bar along the *y*-axis and *z*-axis and $\theta_{y}$ and $\theta_{z}$ are the rotation angles of the boring bar on the *x*- and *x-z* planes.

Substituting Equations (13) and (14) into the strain energy and stress equations, the strain energy of the boring bar can be expressed as

(15) $$\Pi =\frac{1}{2}\int_{L}A_{11}({v^{\prime \prime }}^{2}+{w^{\prime \prime }}^{2})dx$$

where

(16) $$A_{11}=\frac{\pi }{4}\sum_{i=1}^{N}{\overset{–}{K}}_{11i}(R_{i}^{4}-R_{i-1}^{4})$$

In which $R_{i-1}$ and $R_{i}$ are the inner diameter and outer diameter of the *i*-th layer. $R_{i-1}$ and $R_{i}$ are also the functions of the section position *x*.

### 2.3. Kinetic Energy of the Boring Bar

**Figure 2:**
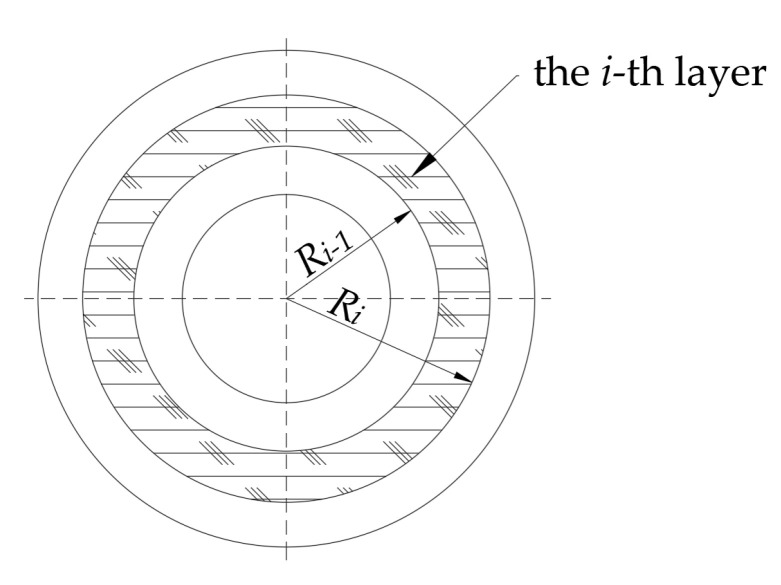
Illustration of the inner and outer radii on the *i*-th layer in the composite boring bar.

Taking both the deformation and rotation of the boring bar into account, the kinetic energy of the boring bar in the global coordinate system can be written as

(17) $$T=\frac{1}{2}\int_{0}^{L}[I_{m}({\dot{v}}^{2}+{\dot{w}}^{2})+I_{d}({\dot{\theta }}_{y}{}^{2}+{\dot{\theta }}_{z}{}^{2})-2I_{P}\Omega \theta_{y}{\dot{\theta }}_{z}+I_{P}\Omega^{2}+\Omega^{2}I_{d}(\theta_{y}{}^{2}+\theta_{z}{}^{2})]dx$$

where $I_{m}$ denotes the mass of the boring bar per unit length and $I_{d}$ and $I_{P}$ denote the inertia moment and the polar inertial moment of the cross-section, which can be expressed as

(18) $$\{\begin{array}{l} I_{m}=\pi \sum_{i=1}^{n}\rho_{i}(R_{i}^{2}-R_{i-1}^{2})\\ I_{d}=\frac{\pi }{4}\sum_{i=1}^{n}\rho_{i}(R_{i}^{4}-R_{i-1}^{4})\\ I_{P}=\frac{\pi }{2}\sum_{i=1}^{n}\rho_{i}(R_{i}^{4}-R_{i-1}^{4}) \end{array}$$

where *n* denotes the total number of plies and $\rho_{i}$ denotes the mass density of the *i*-th layer, as shown in Figure 2.

Substituting the relationship between the rotation angle and the deflection into Equation (17), it can be seen that

(19) $$T=\frac{1}{2}\int_{0}^{L}[I_{m}({\dot{v}}^{2}+{\dot{w}}^{2})+I_{d}({\dot{w}}^{\prime }{}^{2}+{\dot{v}}^{\prime }{}^{2})-2I_{P}\Omega {\dot{w}}^{\prime }{\dot{v}}^{\prime }+I_{P}\Omega^{2}+\Omega^{2}I_{d}({w^{\prime }}^{2}+{v^{\prime }}^{2})]dx$$

### 2.4. Dissipated Virtual Work of the Boring Bar

The dissipated virtual work of the boring bar can be expressed as:

(20) $$\delta W=\int_{V}\sigma_{xx}^{d}\delta \varepsilon_{xx}dV$$

Substituting the expressions of $\sigma_{xx}^{d}$ and $\varepsilon_{xx}$ into Equation (20), the dissipated virtual work of the boring bar can be expressed as

(21) $$\delta W=\int_{L}A_{11}^{\psi }({\dot{v}}^{\prime \prime }\delta v^{\prime \prime }+{\dot{w}}^{\prime \prime }\delta w^{\prime \prime })+A_{11}^{\psi }\Omega (v^{\prime \prime }\delta w^{\prime \prime }-w^{\prime \prime }\delta v^{\prime \prime })dx$$

where

(22) $$A_{11}{}^{\psi }=\frac{\pi }{4}\sum_{i=1}^{N}{\overset{–}{K}}_{11i}{}^{\psi }(R_{i}^{4}-R_{i-1}^{4})$$

### 2.5. Vibration Control Equation of the Boring Bar

Based on the Hamilton principle, the relationship between strain energy density, kinetic energy density, and the dissipated virtual work can be obtained as

(23) δ(T−Π)+δW=0

Substituting the above relation into the Hamilton principle, the vibration differential equation of the system can be expressed as

(24a) $$\begin{array}{l} -I_{m}\ddot{v}+2I_{d}{\ddot{v}}^{\prime \prime }\underline{+2I_{d}{}^{\prime }{\ddot{v}}^{\prime }}+2I_{P}\Omega {\dot{w}}^{\prime \prime }\underline{+2I_{P}{}^{\prime }\Omega {\dot{w}}^{\prime }}\underline{-A_{11}{}^{\prime \prime }}\underline{v^{\prime \prime }-2A_{11}{}^{\prime }v^{\prime \prime \prime }}-A_{11}v^{\prime \prime }{}^{\prime \prime }-\Omega^{2}I_{d}v^{\prime \prime }\underline{-\Omega^{2}I_{d}{}^{\prime }v^{\prime }}+\\ \underline{\underline{\;A_{11}^{\psi }{}^{\prime \prime }{\dot{v}}^{\prime \prime }+2A_{11}^{\psi }{}^{\prime }{\dot{v}}^{\prime \prime \prime }}}+A_{11}^{\psi }{\dot{v}}^{\prime \prime }{}^{\prime \prime }\underline{\underline{-A_{11}^{\psi }{}^{\prime \prime }\Omega w^{\prime \prime }-2A_{11}^{\psi }{}^{\prime }\Omega w^{\prime \prime \prime }}}-A_{11}^{\psi }\Omega w^{\prime \prime }{}^{\prime \prime }=0 \end{array}$$

(24b) $$\begin{array}{l} -I_{m}\ddot{w}+2I_{d}{\ddot{w}}^{\prime \prime }\underline{+2I_{d}{}^{\prime }{\ddot{w}}^{\prime }}-2I_{P}\Omega {\dot{v}}^{\prime \prime }\underline{-2I_{P}{}^{\prime }\Omega {\dot{v}}^{\prime }-A_{11}{}^{\prime \prime }w^{\prime \prime }-2A_{11}{}^{\prime }w^{\prime \prime \prime }}-A_{11}w^{\prime \prime }{}^{\prime \prime }-\Omega^{2}I_{d}w^{\prime \prime }\underline{-\Omega^{2}I_{d}{}^{\prime }v^{\prime }}+\\ \underline{\underline{A_{11}^{\psi }{}^{\prime \prime }{\dot{w}}^{\prime \prime }+2A_{11}^{\psi }{}^{\prime }{\dot{w}}^{\prime \prime \prime }}}+A_{11}^{\psi }{\dot{w}}^{\prime \prime }{}^{\prime \prime }\underline{\underline{+A_{11}^{\psi }{}^{\prime \prime }\Omega v^{\prime \prime }+2A_{11}^{\psi }{}^{\prime }\Omega v^{\prime \prime \prime }}}+A_{11}^{\psi }\Omega v^{\prime \prime }{}^{\prime \prime }=0 \end{array}$$

In above equations, the single-underlined terms indicate the effect of the sectional change on the vibration, and the double-underlined terms indicate the effects of both damping and the sectional change.

### 2.6. Discrete Solution to Vibration Equation

Using the Galerkin approximate solution method, which is commonly used for continuous system vibration and stability analysis, the partial differential equation of free vibration can be transformed into the ordinary differential equation (ODE) with respect to time.

For the cantilever bar, assuming the bending deformation can be expressed as

(25) $$w=\sum_{j=1}^{N}W_{j}(t)\xi_{j}(x),\;\;\;\;\;v=\sum_{j=1}^{N}V_{j}(t)\xi_{j}(x)$$

The vibration mode functions of the bending deformation of standard non-rotary, non-coupling, and uniform cantilever beams are as follows:

(26) $$\begin{array}{l} \cos(\;\beta_{j})\cos h(\beta_{j})=-1,\;\;\;\;\;\;\lambda_{j}=-\frac{\cos\beta_{j}+\cosh\beta_{j}}{\sin\beta_{j}+\sinh\beta_{j}},\;\;\;\;\;\;j=1,2,\cdots N\\ \xi_{j}(x)=\cos(\frac{\beta_{j}x}{L})-\cosh(\frac{\beta_{j}x}{L})+\lambda_{j}(\sin(\frac{\beta_{j}x}{L})-\sinh(\frac{\beta_{j}x}{L})) \end{array}$$

Substituting Equation (25) into the vibration differential equation, the weighted integral was performed on the vibration mode functions using the Galerkin method and then the 2N ordinary differential equations of the boring bar can be obtained as

(27) $$M\left\{\ddot{X}\right\}+G\{\dot{X}\}+K\left\{X\right\}=0$$

where

(28) $$K=\left[\begin{array}{cc} K_{11ij} & K_{12ij}\\ K_{21ij} & K_{22ij} \end{array}\right],M=\left[\begin{array}{cc} M_{11ij} & 0\\ 0 & M_{22ij} \end{array}\right],G=\left[\begin{array}{cc} G_{11ij} & G_{12ij}\\ G_{21ij} & G_{22ij} \end{array}\right],X={\left(\begin{array}{cc} \sum_{j=1}^{N}V_{j}(t) & \sum_{j=1}^{N}W_{j}(t) \end{array}\right)}^{T}\;\begin{array}{l} M_{11ij}=\int_{0}^{L}(-I_{m}\xi_{i}+2I_{d}\xi_{i}{}^{\prime \prime }+2I_{d}{}^{\prime }\xi_{i}{}^{\prime })\xi_{j}dx,\;\;\;M_{22ij}=M_{11ij},\\ K_{11ij}=\int_{0}^{L}(-A_{11}{}^{\prime \prime }\xi_{i}{}^{\prime \prime }-2A_{11}{}^{\prime }\xi_{i}{}^{\prime \prime \prime }-A_{11}{\xi_{i}}^{\prime \prime }{}^{\prime \prime })\xi_{j}dx,\;\;\;K_{22ij}=K_{11ij}\\ K_{12ij}=\int_{0}^{L}(-A_{11}^{\psi }{}^{\prime \prime }\Omega \xi_{i}{}^{\prime \prime }-2A_{11}^{\psi }{}^{\prime }\Omega \xi_{i}{}^{\prime \prime \prime }-A_{11}^{\psi }\Omega {\xi_{i}}^{\prime \prime }{}^{\prime \prime })\xi_{j}dx,\;\;\;K_{21ij}=-K_{12ij}\\ G_{11ij}=\int_{0}^{L}(A_{11}^{\psi }{}^{\prime \prime }\xi_{i}{}^{\prime \prime }+2A_{11}^{\psi }{}^{\prime }\xi_{i}{}^{\prime \prime \prime }+A_{11}^{\psi }{\xi_{i}}^{\prime \prime }{}^{\prime \prime })\xi_{j}dx,\;\;\;G_{22ij}=G_{11ij}\\ G_{12ij}=\int_{0}^{L}(2I_{P}\Omega \xi_{i}{}^{\prime \prime }+2I_{P}{}^{\prime }\Omega \xi_{i}{}^{\prime })\xi_{j}dx,\;\;\;G_{21ij}=-G_{12ij} \end{array}$$

From Equation (28), it can be seen that the non-diagonal terms in the damping matrix G reflect the influence of the gyroscopic effect, and the diagonal items reflect the influence of the material resistance. The vortex motion appeared when the boring bar was influenced by the gyroscopic effect.

## 3. Boring Stability Analysis

**Figure 3:**
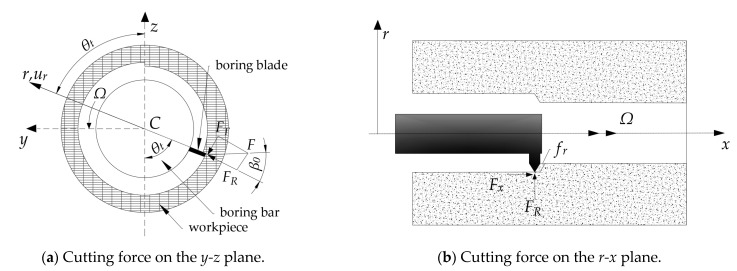
Model of the cutting force in the boring.

This study adopted the model of boring force established by Kapoor et al., as shown in Figure 3 [20]. The boring force is in direct proportion to the cutting cross-sectional area on the *r-x* plane. Assuming that the endpoint of the bar *C* shows no displacement on the *y-z* plane, the boring force *F* can be expressed as

(29) $$F=K_{s}f_{r}d_{r}$$

where $K_{s}$ denotes the cutting force on the unit cutting area; $f_{r}$ denotes the feed per rotation during the boring process; and $d_{r}$ denotes the cutting radial depth. During the boring process, due to the deformation of the boring bar, the endpoint *C* will deviate along the radial depth direction. Assuming that $u_{r}$ denotes the offset, the boring force after the consideration of the deviation can be expressed as

(30) $$F=K_{S}f_{T}(d_{T}-u_{T})$$

In combination with Equation (29), the cutting force in Figure 3a can be obtained as

(31) $$F_{T}(t)=F(t)\sin\beta_{0};\;\;\;F_{R}(t)=F(t)\cos\beta_{0}$$

where $\beta_{0}$ denotes the intersection angle between the normal direction of the cutting face and the *y-z* plane and $\beta_{0}=60{}^{\circ}$ [20]. With reference to the research results by Subramani et al. [21], the axial cutting force can be assumed as

(32) $$F_{x}(t)=-0.5F_{T}(t)$$

From Figure 3, through the transformation of coordinates, the boring forces along the *y*-axis and *z*-axis can be obtained as

(33) $$\begin{array}{l} F_{y}(t)=F_{y0}(t)-F_{y1}(t)[v(t)-v(t-\tau )]-F_{y2}(t)[w(t)-w(t-\tau )]\\ F_{z}(t)=F_{z0}(t)-F_{z1}(t)[v(t)-v(t-\tau )]-F_{z2}(t)[w(t)-w(t-\tau )] \end{array}$$

where

(34) $$\begin{array}{l} F_{z0}(t)=K_{s}f_{r}d_{r}\cos(\theta_{t}+\beta_{0})\\ F_{z1}(t)=K_{s}f_{r}\{0.5\cos\beta_{0}+0.5\cos(2\theta_{t}+\beta_{0})\}\\ F_{z2}(t)=K_{s}f_{r}\{-0.5\sin\beta_{0}+0.5\sin(2\theta_{t}+\beta_{0})\}\\ F_{y0}(t)=K_{s}f_{r}d_{r}\sin(\theta_{t}+\beta_{0})\\ F_{y1}(t)=K_{s}f_{r}\{0.5\sin\beta_{0}+0.5\sin(2\theta_{t}+\beta_{0})\}\\ F_{y2}(t)=K_{s}f_{r}\{0.5\cos\beta_{0}-0.5\cos(2\theta_{t}+\beta_{0})\} \end{array}$$

In which $\theta_{t}=\Omega t$ denotes the angular displacement during the rotation of the boring bar; $K_{s}$ is the coefficient of cutting force during high-speed boring on steel; according to the test data in [20], $K_{s}=1330\times 10^{6}$ N/m^2^; and $f_{r}$ denotes the boring feed, with the unit of mm/rotation.

During single-tool boring machining, the boring force refers to the stress imposed on the endpoint at *x* = *L* of the boring bar. Combined with the research results in refs. [22] and [23], and assuming that $f_{y}$ and $f_{z}$ denote the cutting forces along the *y*-axis and *z*-axis direction per unit length, respectively, Equation (27) can be rewritten as

(35) $$M\left\{\ddot{X}\right\}+G\{\dot{X}\}+K\left\{X\right\}=F$$

where

(36) $$F=\left\{\begin{array}{c} f_{i}^{\left(y\right)}\\ f_{i}^{\left(z\right)} \end{array}\right\};f_{i}^{\left(y\right)}=\int_{0}^{L}f_{y}\xi_{i}dx;f_{i}^{\left(z\right)}=\int_{0}^{L}f_{z}\xi_{i}dx$$

(37) $$\begin{array}{l} f_{y}=F_{y}(t)\delta (x-L),\;\;\;0\leq x\leq L\\ f_{z}=F_{z}(t)\delta (x-L),\;\;\;0\leq x\leq L \end{array}$$

In which δ is the Dirac-delta function and can be expressed as

(38) $$\delta (x)=\{\begin{array}{c} \infty ,\;\;\;x=0\\ 0,\;\;\;x\neq 0 \end{array}$$

Substituting Equations (36)–(38) into Equation (35) and combined with Equation (25), the vibration equation of the boring bar imposed by cutting forces can be obtained as

(39) $$M\ddot{X}(t)+G\dot{X}(t)+KX(t)+K_{F}\{X(t)-X(t-T)\}=f_{0i}$$

where **M**, **G**, and **K** are identical to those in Equation (28). The other parameters are as follows:

(40) $$\begin{array}{l} X(t)=\left\{\begin{array}{c} v(t)\\ w(t) \end{array}\right\};\;\;f_{0i}=\left\{\begin{array}{c} f_{0y}\\ f_{0z} \end{array}\right\};\;\;f_{0y}=F_{y0}\{\xi_{i}(L)\};\;\;f_{0z}=F_{z0}\{\xi_{i}(L)\}\\ K_{F}=K_{f}+K_{p}(t);\;\;\;\;K_{f}=\frac{K_{s}f_{r}}{2}\left[\begin{array}{cc} \cos\beta_{0}X & -\sin\beta_{0}X\\ \sin\beta_{0}X & \cos\beta_{0}X \end{array}\right];\\ K_{p}(t)=\frac{K_{s}f_{r}}{2}\left[\begin{array}{cc} \cos(2\theta_{t}+\beta_{0})X & \sin(2\theta_{t}+\beta_{0})X\\ \sin(2\theta_{t}+\beta_{0})X & -\cos(2\theta_{t}+\beta_{0})X \end{array}\right];\;\;\;X_{ij}=\xi_{i}(L)\xi_{j}(L) \end{array}$$

According to Equation (40), the off-diagonal elements in the $K_{f}$ matrix are opposite in sign, which can induce chattering instability during the cutting process. The elements in the $K_{f}$ matrix change in a periodic pattern, which can also induce unstable boring parameters.

During the boring process, a constant force will not induce the chatter of the boring bar. Accordingly, the constant force on the right side of Equation (39) can be ignored. Only considering the effect of the time-delay force on the boring bar, the boring vibration equation can be expressed as

(41) $$M\ddot{X}(t)+G\dot{X}(t)+(K+K_{F})X(t)=K_{F}X(t-T)$$

Equation (41) is a standard 2-dof delay differential equation during the cutting vibration. The time-delay terms related to the bending and deformation of the boring bar can induce chattering.

## 4. Analysis of Numerical Results

### 4.1. Natural Vibration Analysis

The first-order inherent frequency of the boring bar imposes great significance on the cutting stability. In this study, in order to reveal the effects of the various factors on the boring bar’s inherent vibration characteristics, the inherent vibration characteristics of the hollow circular-section boring bar made up of epoxy-resin-based graphite fibers were analyzed. The boring bar is shown in Figure 1, and Table 1 lists the performance parameters of the material. From the table, *N* = 10, $t_{c}=0.01321\;m$, $d=2R=r_{1}+r_{2}=0.127\;m$, $\psi_{1}$ = 0.0045, $\psi_{2}$ = 0.0422, and $\psi_{12}$ = 0.0705, where *N* denotes the number of plies, whereas $\psi_{1}$, $\psi_{2}$, and $\psi_{12}$ are the internal damping parameters. For convenience for the subsequent description, we used ψ = 1 to represent this group of values and used ψ = 0.5 to represent the parameters of $\psi_{1}$ = 0.00225, $\psi_{2}$ = 0.0211, and $\psi_{12}$ = 0.03525. The rest were treated in the same manner. If there was no specified explanation, the boring bars all adopted the [0°]_10_-ply mode. The taper ratio (TR) is to reveal the linear sectional change of the boring bar, which can be defined as

(42) $$\mathrm{TR}=\frac{(r_{1}-r_{2})\times 100}{L},\;\;\;r_{1}\geq r_{2}$$

**Figure 4:**
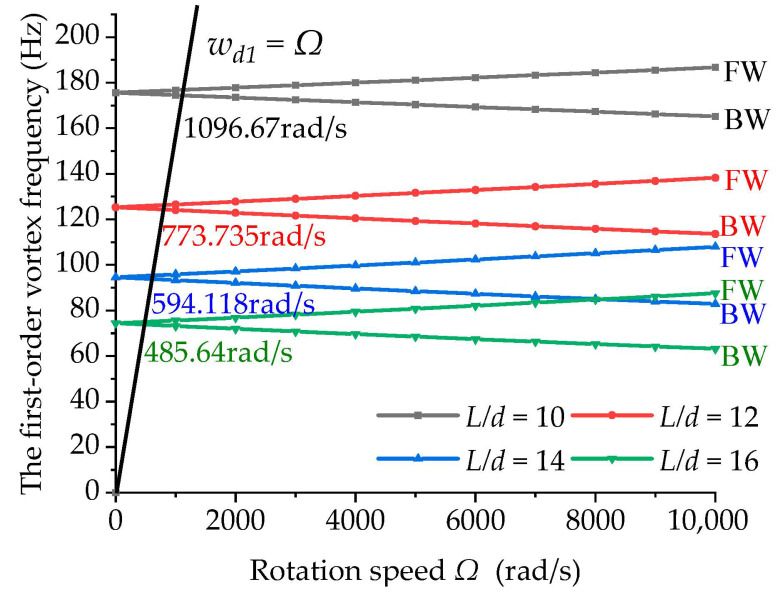
Effect of the length-to-diameter ratio on the first-order vortex frequency (*TR* = 1 and ψ=0).

Figure 4 shows the variation in the first-order vortex frequency of the boring bars with different length-to-diameter ratios when *TR* = 1 and ψ = 0, in which FW and BW denote the forward and backward procession, respectively. In the figure, it can be seen that as the length-to-diameter ratio increased, the first-order vortex frequency decreased gradually, and simultaneously, the critical rotation speed decreased. As the length-to-diameter ratio increased, the boring bar became thinner and longer, thereby reducing the first-order natural bending frequency and the critical rotation speed. Accordingly, the chattering was more likely to occur.

**Figure 5:**
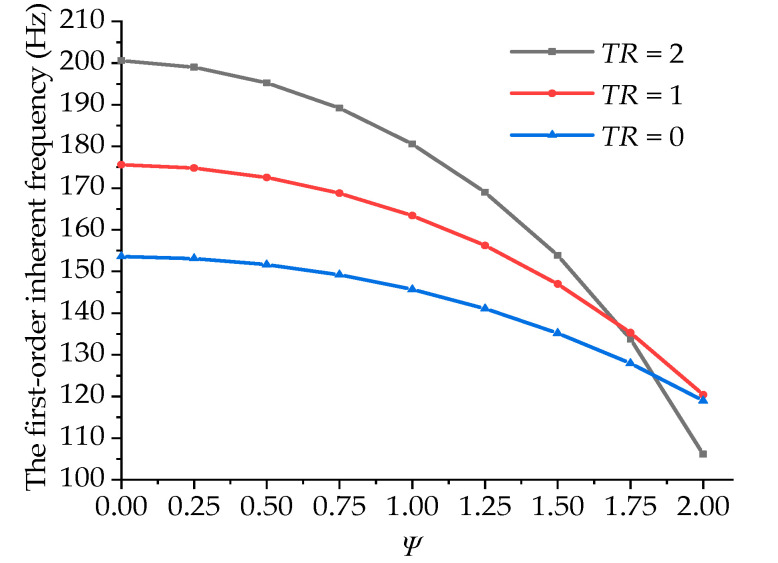
Variations in the first-order inherent frequency with the material damping of the boring bar under different taper ratios (*L/d* = 10).

Figure 5 displays the variation in the first-order inherent frequency with the internal damping coefficient under a fixed length-to-diameter ratio and a changing taper ratio. As the internal damping coefficient increased, the first-order inherent frequency of the boring bar decreased gradually, and the larger the taper ratio, the more significant the effect influenced by the inner damping of the boring bar. This can be attributed to the existence of the double-underlined terms as shown in Equation (24) in the vibration equation. A larger taper ratio is indicative of a greater effect on the change in damping.

**Figure 6:**
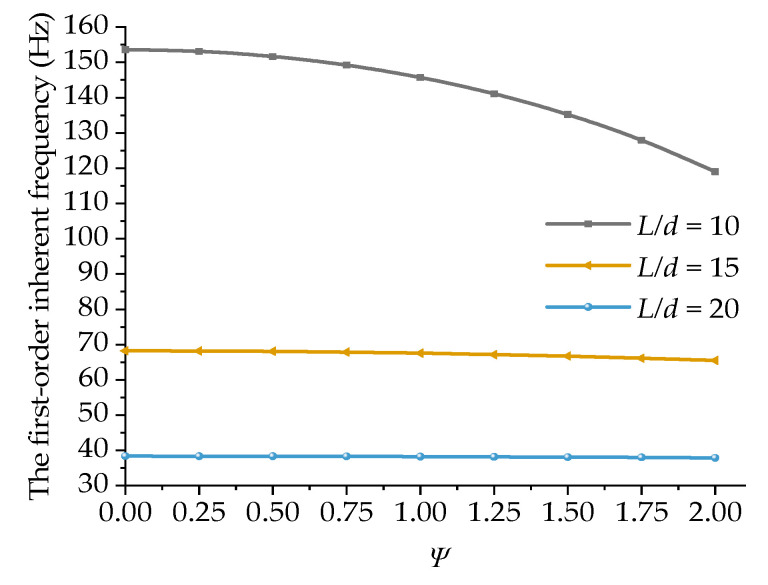
Variations in the first-order inherent frequency with the damping ratio of the boring bar under different length-to-diameter ratios (*TR* = 0 and ψ=0).

Figure 6 shows the changes in the inherent frequency of uniform-section boring bars with different length-to-diameter ratios with the damping coefficient. For the boring bars with different length-to-diameter ratios, the inherent frequency decreased gradually with the increase in the damping coefficient, and the smaller the length-to-diameter ratio, the more significant the effect of the internal damping coefficient on the first-order inherent frequency of the boring bar. This suggests that the effect of $A_{11}^{\psi }$ in Equation (24) was weakened for thinner and longer bars. Accordingly, the effect of the inherent frequency on damping weakened and the curve flattened. For the boring bars with large length-to-diameter ratios, changing the inherent frequency with damping showed a limited effect.

**Figure 7:**
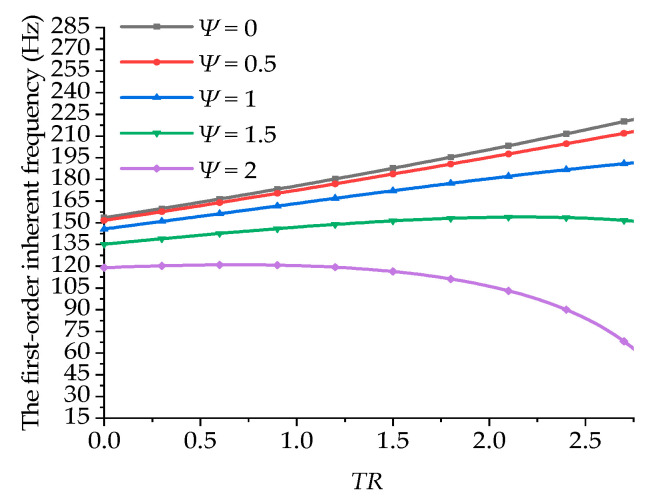
Effect of the taper ratio of the boring bar on the first-order inherent frequency (ψ=0 and *L/d* = 10).

Figure 7 shows the variations in the first-order inherent frequencies of the boring bars with the taper ratio under a fixed length-to-diameter ratio and different damping coefficients. In the figure, it can be seen that when the damping ratio equaled 0, 0.5, and 1, the first-order inherent frequency of the boring bar increased gradually with the increasing taper ratio. When the damping ratio equaled 1.5 and 2, the inherent frequency first increased and then decreased with the increase in the taper ratio. This can also be explained by the existence of the double-underlined terms in Equation (24). The inherent frequency of the boring bar with low damping can be enhanced by increasing the taper ratio. For the materials with large damping, the taper ratio imposed a complex effect. The effects of both the taper ratio and damping should be considered.

**Figure 8:**
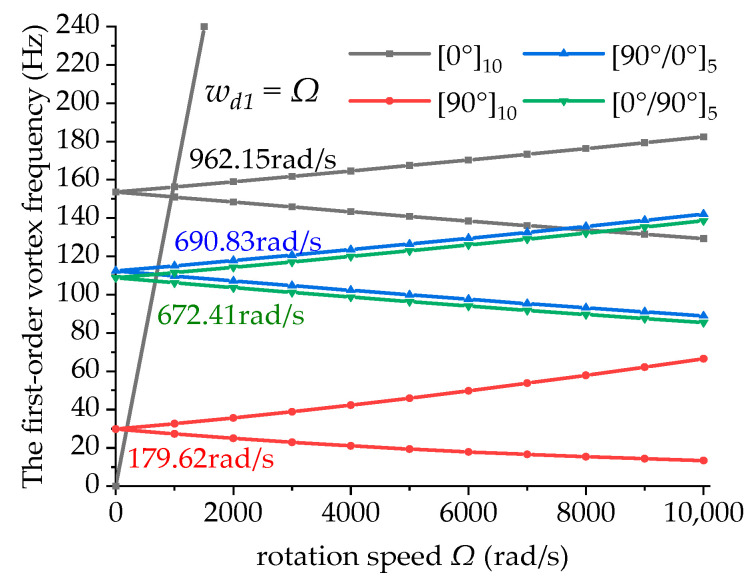
Effect of the ply mode on the first-order vortex frequency of the boring bar (ψ=0, *L/d* = 10 and *TR* = 0).

Figure 8 shows the variations in the first-order vortex frequency of the boring bars with the rotation speed under different ply modes. The definitions of BW and FW are consistent with those in previous figures and are not repeated here. In the figure, it can be seen that the greater the number of 0° ply layers, the greater the first-order vortex frequency and the larger the critical rotation speed of the boring bars. Moreover, the critical rotation speed of the boring bar decreased with the decreasing number of 0° ply layers. The first-order vortex frequency of the material with a ply mode of [90°]_10_ was minimum. Through comparison, the first-order vortex frequency of the bar with a ply mode of [90°/0°]_5_ was slightly higher than that of the bar with a ply mode of [0°/90°]_5_. This suggests that the closer the 0° ply to the outer surface of the boring bar, the higher the critical rotation speed. This is because the 0° ply showed higher rigidity than the plies with other angles. Meanwhile, the cross-section where the 0° ply was located was greater in radius, perimeter, and volume, and thus can bear greater the bending moment induced by elastic stress and dissipative stress. Therefore, compared with the arrangement of 0° ply on the inner layer, arranging the 0° ply on the outer layer can enhance the stability of the boring bar.

**Figure 9:**
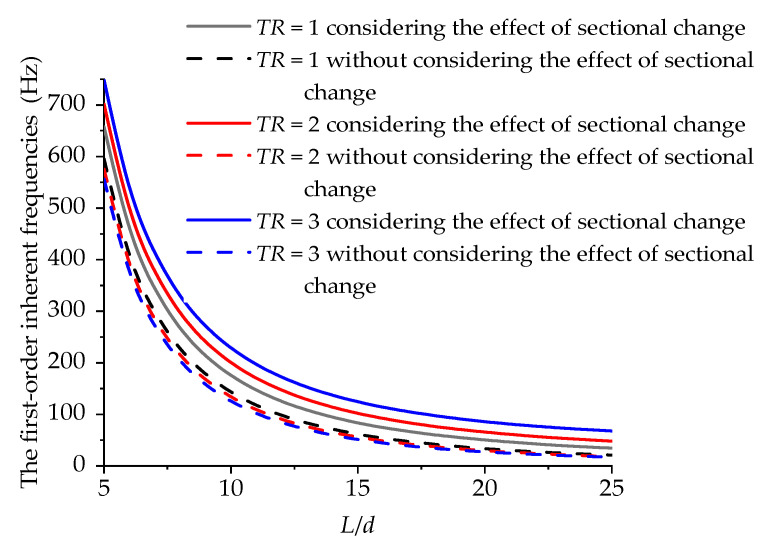
Variations in the first-order inherent frequencies of the boring bar with the length-to-diameter ratio under different *TR* values (ψ=0).

**Figure 10:**
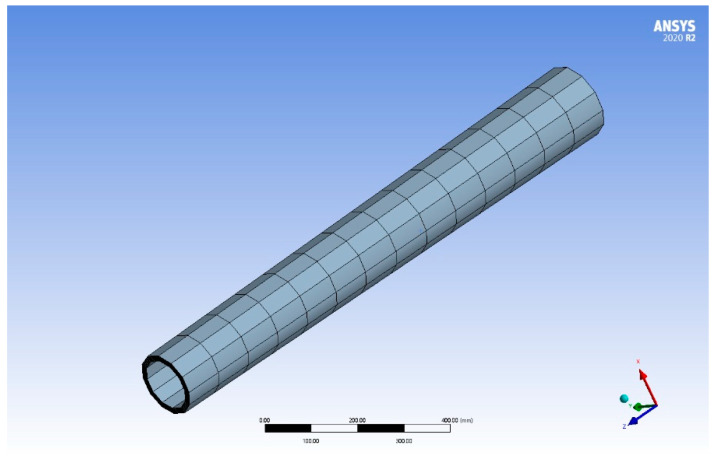
Schematic diagram of composite toolholder model (ψ=0, *TR* = 1, *L/d* = 12).

Compared with existing models, the established stability analysis model of the variable-stability boring bar considered the effect of the sectional change. In order to analyze the sectional change in the inherent vibration characteristics, the calculation results before and after the consideration of the sectional change were compared for further analysis. Since both $A_{11}^{\psi }{}^{\prime }$ and $A_{11}^{\psi }{}^{\prime \prime }$ included the effects of damping on the boring bar’s inherent vibration characteristics, the internal damping coefficient was set as 0 only to analyze the effect of the sectional change on the boring bar’s vibration characteristics. Then, the free vibration characteristics were analyzed by setting the terms of $I_{d}{}^{\prime }$, $I_{P}{}^{\prime }$, $A_{11}{}^{\prime }$, and $A_{11}{}^{\prime \prime }$ in Equations (24a) and (24b) as 0. Figure 9 shows the effect of the sectional change on the first-order inherent frequency of the boring bar when *TR* = 1, 2, and 3, respectively. The larger the taper ratio of the boring bar, the greater the effect. Table 2 lists the calculation results of the first-order inherent frequencies of the boring bar with different *TR* values using different models. The ANSYS model of the boring bar is shown in Figure 10. Solid 185 element is used to simulate the composite laminated structure. From the table, the calculation results using the established model were closer to the data using the ANSYS model, showing more favorable reliability.

### 4.2. Cutting Stability Analysis

#### Validation of Model Reliability

**Figure 11:**
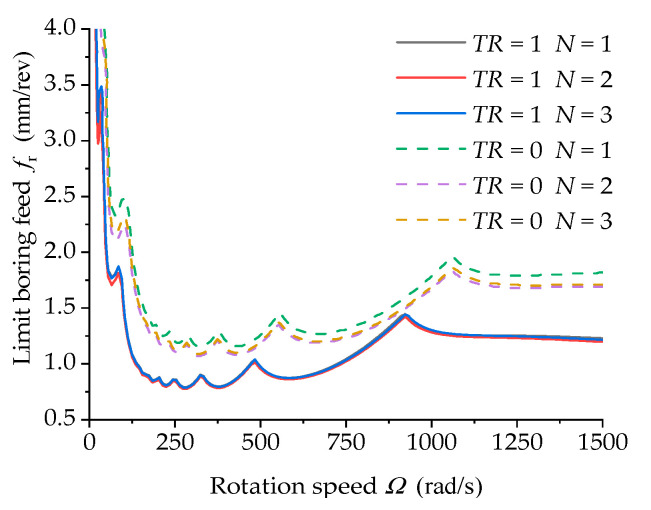
Effect of the number of vibration mode functions on the stability lobe diagrams of the rotated composite boring bar (ψ=1 and *L/d* = 10).

The Galerkin method is a numerical discrete algorithm, whose numerical calculation results are related to precision and the number of intercepted modes. In the case of insufficient intercepted modes, the calculation results cannot be convergent with great errors. However, a larger number of intercepted modes is also not good. In the case of a larger number of modes, the calculation time is long and the calculation results are divergent. Therefore, it is necessary to validate the convergence of the selected number of modes used in this study. Figure 11 shows the calculated stability lobes of different boring bars with different mode numbers. In the figure, it can be seen that the stability lobe diagrams were quite similar when the model numbers were set as 1, 2, and 3. Table 3 shows the limit feeds of the unconditional stable region at a rotation speed of 0~1500 rad/s using different mode numbers. The numerical results were quite close, suggesting the favorable convergence of the solution to the stability analysis of the boring system using the time-domain semi-discrete method.

**Figure 12:**
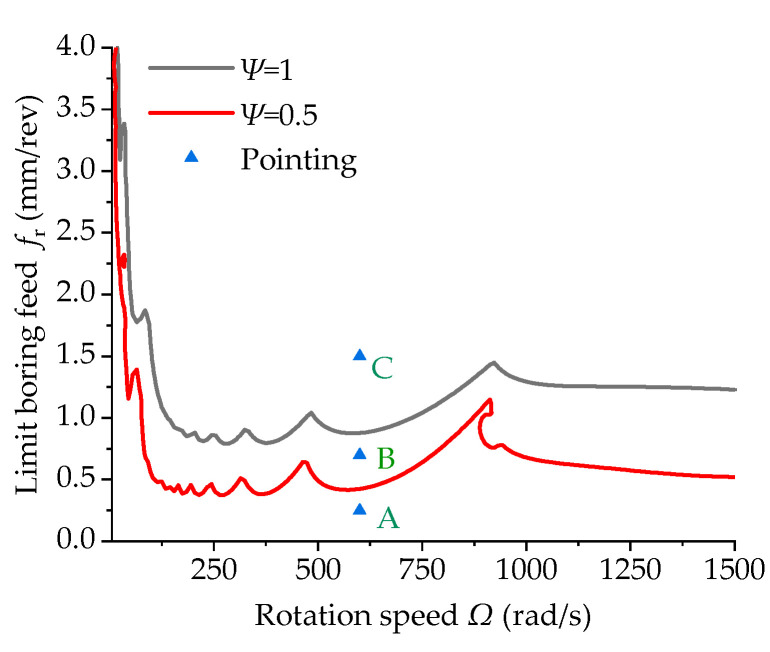
Stability lobe diagrams of the boring bar with different internal damping coefficients (*L/d* = 10 and *TR* = 0).

**Figure 13:**
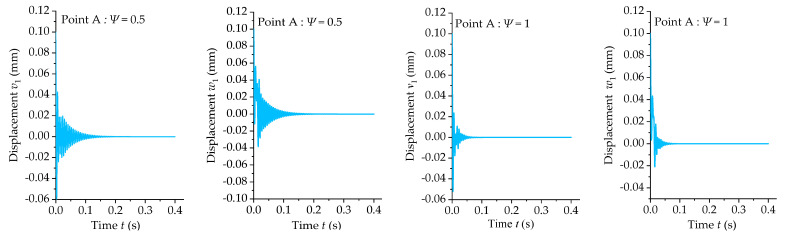

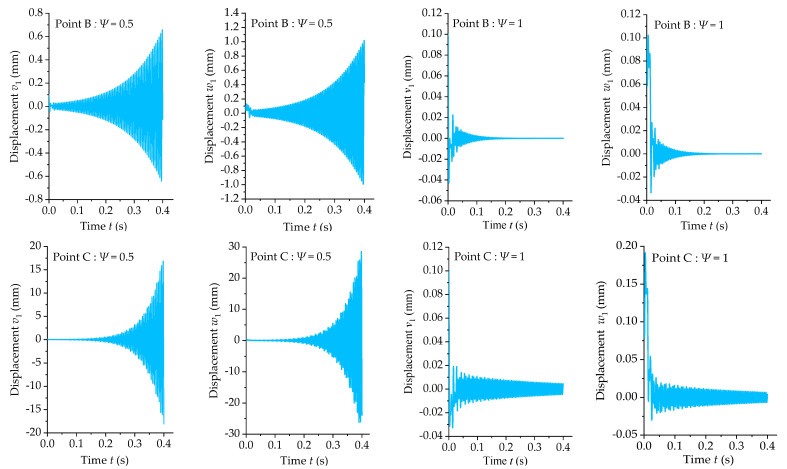
Displacement response curves of Points A, B, and C.

Cutting stability analysis curves can be obtained via feature extraction. It is necessary to validate the reliability of cutting stability. This study used the Runge–Kutta method to perform the displacement response analysis on the control equation and validated the results by combining the displacement response results and stability analysis curves. Figure 12 shows the cutting stability lobes of the uniform section of the boring bars with different damping coefficients. A total of 3 points, denoted as Point A, Point B, and Point C, were selected for the displacement response analysis, and the results are shown in Figure 13.

Table 4 lists the rotation speeds and feeds at different coordinates. These points were located in the stable and unstable regions of the lobe diagrams of the boring bars with different damping coefficients. In Figure 12 it can be seen that Point A was located in the stable regions of the boring bars with damping coefficients of 0.5 and 1, indicating its displacement response should be convergent. These results were consistent with the results shown in Figure 13. Point B was located in the unstable region of the boring bar with a damping coefficient of 0.5 and the stable region of the boring bar with a damping coefficient of 1, indicating its displacement response should be divergent and convergent. These results were also consistent with the divergent and convergent displacement responses at Point B in Figure 13. Point C was located in the unstable regions with damping coefficients of 0.5 and 1, indicating its displacement response should be divergent. These results were consistent with the divergent displacement response at Point C in Figure 13. The displacement response results fit well with the conclusions drawn from the lobe diagrams, suggesting the reliability of the lobe diagrams.

### 4.3. Analysis of the Influencing Factors of the Boring Stability of the Boring Bar

**Figure 14:**
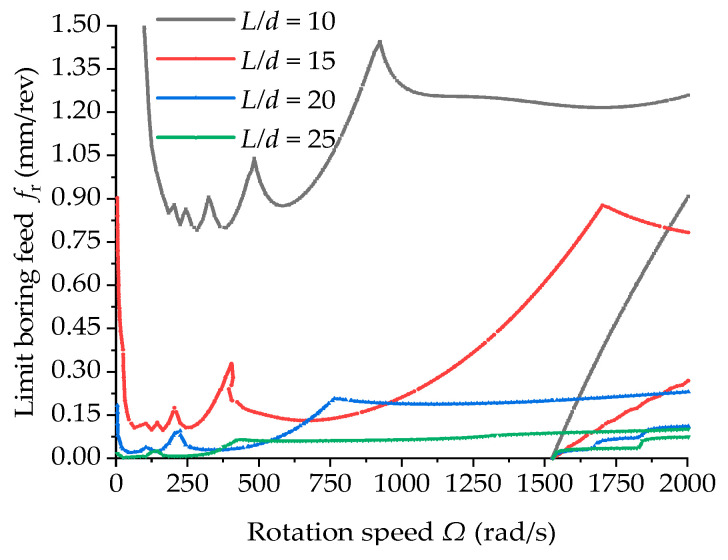
Stability lobe diagrams of the boring bar with different length-to-diameter ratios (ψ=1 and *TR* = 0).

Figure 14 shows the boring stability lobe diagrams of the boring bars with different length-to-diameter ratios. In the figure it can be seen that the limit feed of the boring bar at the same rotation speed decreased with the increasing length-to-diameter ratio, suggesting that the increase in the length-to-diameter ratio increased the instability. When the length-to-diameter ratio exceeded 10, the limit cutting depth dropped rapidly, indicating great difficulties in deep-hole processing. A greater length-to-diameter ratio is indicative of thinner pores. At that moment, the boring bar with a larger length-to-diameter ratio is featured by quite low rigidity with a very high risk of chattering. The limit cutting depth is quite low with a fairly low processing efficiency. In addition, it can also be observed from the figure that the length-to-diameter ratio of the boring bar imposed a slight effect on the initiation speed of new chatter instability in the high-speed rotation region.

**Figure 15:**
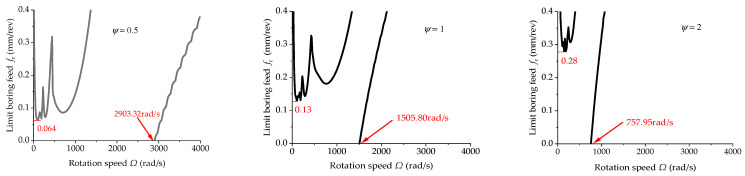
Effect of the change in internal damping on the stability (*L* = 2 m and *TR* = 1).

Figure 15 shows the effect of the change in internal damping on the cutting stability of the boring bar when *TR* = 1 and *L* = 2 m (*L*/*d* = 15.7). Through comparison, when the rotation speed was low, increasing the internal damping of the boring bar can enhance the cutting stability of the boring bar, thereby leading to greater stable feed. However, at a high rotation speed, a new boring chatter unstable region appeared due to the existence of damping. The greater the damping, the smaller the initiation speed of the unstable region and the larger the range of the unstable region enclosed by the high-speed lobe curve and the horizontal axis. Generally, a higher inherent frequency suggests a larger limit cutting depth, whereas the inner damping shows an opposite effect. Under higher inner damping, the inherent vibration frequency can be reduced but still enhance the cutting stability at a low rotation speed, thereby reducing the cutting stability at a high rotation speed.

**Figure 16:**
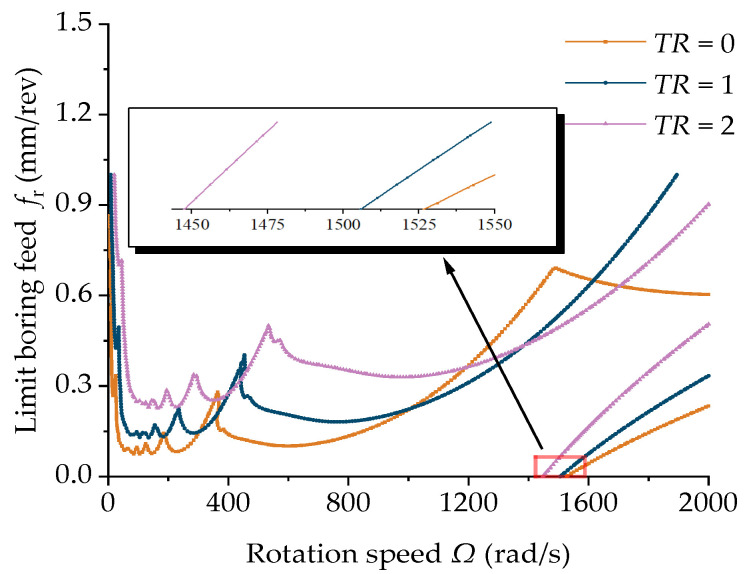
Effect of the taper ratio on the stability of the boring bar (ψ=1 and *L =* 2 m).

Figure 16 shows the effect of the taper ratio of the boring bar on the cutting stability. From the figure it can be seen that ψ=1 at the same rotation speed and increasing the taper ratio of the boring bar can obtain a greater stable feed, thereby enhancing the cutting efficiency. However, as the taper ratio increased, the initialization speed of the new high-speed chattering unstable region changed slightly. By combining Figure 14, Figure 15 and Figure 16, it can be concluded that the initialization speed of the cutting unstable region at a high rotation speed was most significantly affected by the damping coefficient but slightly affected by the taper ratio. The length-to-diameter ratio imposed almost no effect.

**Figure 17:**
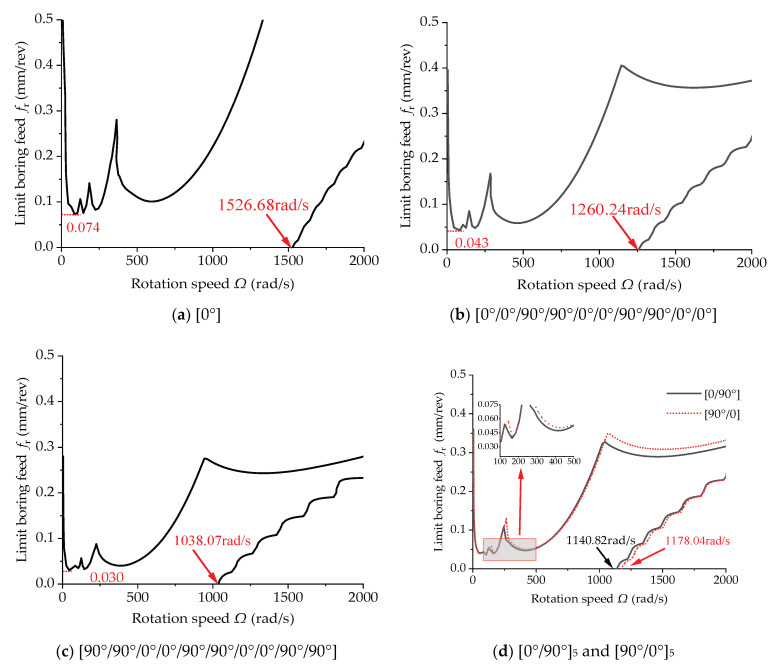
Effect of the ply angle on the stability of the boring bar (ψ=1, *TR* = 0 and *L* = 2 m).

Figure 17 shows the effect of the ply angle on the cutting stability of the uniform-section boring bar with a length of 2 m. Through comparison, as the number of 0° plies decreased and the 90° plies increased, the limit boring feed dropped from 0.074 mm/rev to 0.03 mm/rev and the initialization speed of the new boring chattering unstable region decreased from 1526.68 rad/s to 1038.07 rad/s. It can be concluded that the more 0° plies, the greater the minimum limit feed of the boring bar and the greater the initialization speed of the new boring chattering unstable region.

Figure 17d shows the stability lobe diagrams of the boring bars with ply modes of [0°/90°]_5_ and [90°/0°]_5_, respectively. The limit stable cutting depth increased slightly when 0° plies were arranged from inside to outside. It should be noted that more 0° plies were more favorable for bending deformation; however, the composite boring bar with a ply angle of 0° was the weakest in torsion resistance.

## 5. Conclusions

Aiming at overcoming the limitations in the current variable-section boring bar models, this study introduced both sectional change and inner damping to establish the kinetic control equations of composite boring bars with large length-to-diameter ratios. Moreover, the effects of the various factors on the inherent vibration frequency and cutting stability were qualitatively and quantitatively analyzed in theory. This study innovatively explored the variation rules of the inherent frequency and cutting stability by taking both the sectional change and damping into account. The main conclusions are described below.

(1) The increase in the length-to-diameter ratio can reduce the first-order inherent frequency of free vibration and limit the boring feed while imposing no effect on the initiation speed of the new chattering unstable region.
(2) The increase in the internal damping coefficient can reduce the first-order free vibration inherent frequency of the boring bar and simultaneously enhance the limit boring feed. However, at a high rotation speed, the existence of damping will generate a new boring chattering unstable region. Moreover, the unstable region will appear earlier under a greater damping condition.
(3) At a damping ratio of 1 (i.e., ψ=1), as the taper ratio of the boring bar increased, the first-order inherent frequency and the limit feed during stable boring were enhanced. The taper ratio imposed a slight effect on the initiation speed of the new chattering unstable region during high-speed boring. Meanwhile, both the taper ratio of the boring bar and the damping coefficient had interaction effects on the free vibration characteristics and cutting stability. For the composite variable-section boring bar, the effects of the internal damping and taper ratio should be considered. This is an innovative point in this study.
(4) The ply angle can significantly affect the stable region during the boring process. When the ply angle was 0°, the stable region achieved the maximum, accompanied by large rigidity and small damping. Therefore, the external surface should adopt a 0° ply mode to enhance the cutting stability.

## Figures and Tables

**Table 1:** Performance parameters of epoxy-resin-based graphite fibers.

	Graphite Epoxy	Steel
*E*_1_ (GPa)	192	207
*E*_2_ = *E*_3_ (GPa)	7.24	207
*G*_12_ = *G*_13_ (GPa)	4.07	80
*G*_23_ (GPa)	3	80
*v*_12_ = *v*_13_	0.24	0.2975
*ρ* (kg/m^3^)	1610	7700

**Table 2:** Effect of the change of the length-to-diameter ratio on the first-order inherent frequency of the boring bar under different *TR* values (*ψ* = 0).

Taper Ratio of the Boring Bar	Length-to-Diameter Ratio		First-Order Inherent Frequency (Hz)	
		Simulation Results of the ANSYS Model (AR)	The Established Model Considering the Sectional Change and the Errors Compared with the AR Simulation Results	The Established Model without Considering the Sectional Change and the Errors Compared with the AR Simulation Results
*TR* = 1	10	163.24	175.62 7.58%	143.51 −12.09%
	12	123.05	125.29 1.82%	98.29 −20.12%
	14	96.748	94.56 −2.26%	71.23 −26.38%
*TR* = 2	10	180.45	200.59 11.16%	134.11 −25.68
	12	138.78	146.98 5.91%	90.68 −34.65%
	14	110.83	113.93 2.80%	64.90 −41.44

**Table 3:** Effect of the number of vibration mode functions on the limit boring feed of the rotated composite material boring system (ψ=1 and *L/d* = 10).

Number of Vibration Mode Functions	1	2	3
Limit boring feed when *TR* = 1 (mm/rev)	1.149986	1.074399	1.091239
Limit boring feed when *TR* = 0 (mm/rev)	0.790758	0.776207	0.787462

**Table 4:** Rotation speeds and cutting depths of the different points in Figure 11.

Point	Rotation Speed (rad/s)	Feed (mm/rev)
A	600	0.25
B	600	0.75
C	600	1.5

## Appendix A

(A1) $$\begin{array}{l} K_{11}={\overset{–}{Q}}_{11}\sin^{4}\beta +2({\overset{–}{Q}}_{13}+2{\overset{–}{Q}}_{55})\sin^{2}\beta \cos^{2}\beta +{\overset{–}{Q}}_{33}\cos^{4}\beta ,\\ K_{12}={\overset{–}{Q}}_{12}\sin^{2}\beta +{\overset{–}{Q}}_{23}\cos^{2}\beta ,\\ K_{13}=(\cos^{4}\beta +\sin^{4}\beta ){\overset{–}{Q}}_{13}+({\overset{–}{Q}}_{11}+{\overset{–}{Q}}_{33}-4{\overset{–}{Q}}_{55})\sin^{2}\beta \cos^{2}\beta ,\\ K_{14}=({\overset{–}{Q}}_{16}-2{\overset{–}{Q}}_{45})\cos\beta \sin^{2}\beta +{\overset{–}{Q}}_{36}\cos^{3}\beta ,\\ K_{15}=({\overset{–}{Q}}_{13}-{\overset{–}{Q}}_{11}+2{\overset{–}{Q}}_{55})\sin^{3}\beta \cos\beta +({\overset{–}{Q}}_{33}-{\overset{–}{Q}}_{13}-2{\overset{–}{Q}}_{55})\cos^{3}\beta \sin\beta ,\\ K_{16}=-{\overset{–}{Q}}_{16}\sin^{3}\beta -(2{\overset{–}{Q}}_{45}+{\overset{–}{Q}}_{36})\sin\beta \cos^{2}\beta ,\;\;\;\;K_{22}={\overset{–}{Q}}_{22},\\ K_{23}={\overset{–}{Q}}_{12}\cos^{2}\beta +{\overset{–}{Q}}_{23}\sin^{2}\beta ,\;\;\;\;\;\;\;K_{24}={\overset{–}{Q}}_{26}\cos\beta ,\\ K_{25}=({\overset{–}{Q}}_{23}-{\overset{–}{Q}}_{12})\sin\beta \cos\beta ,\;\;\;\;\;\;\;K_{26}=-{\overset{–}{Q}}_{26}\sin\beta ,\\ K_{33}={\overset{–}{Q}}_{11}\cos^{4}\beta +{\overset{–}{Q}}_{33}\sin^{4}\beta +2({\overset{–}{Q}}_{13}+2{\overset{–}{Q}}_{55})\sin^{2}\beta \cos^{2}\beta ,\\ K_{34}={\overset{–}{Q}}_{16}\cos^{3}\beta +(2{\overset{–}{Q}}_{45}+{\overset{–}{Q}}_{36})\sin^{2}\beta \cos\beta ,\\ K_{35}=({\overset{–}{Q}}_{13}-{\overset{–}{Q}}_{11}+2{\overset{–}{Q}}_{55})\sin\beta \cos^{3}\beta +({\overset{–}{Q}}_{33}-{\overset{–}{Q}}_{13}-2{\overset{–}{Q}}_{55})\sin^{3}\beta \cos\beta ,\\ K_{36}=-{\overset{–}{Q}}_{36}\sin^{3}\beta +(2{\overset{–}{Q}}_{45}-{\overset{–}{Q}}_{16})\sin\beta \cos^{2}\beta ,\\ K_{44}={\overset{–}{Q}}_{44}\sin^{2}\beta +{\overset{–}{Q}}_{66}\cos^{2}\beta ;\;\;\;\;K_{46}=({\overset{–}{Q}}_{44}-{\overset{–}{Q}}_{66})\sin\beta \cos\beta ,\\ K_{45}=-{\overset{–}{Q}}_{45}\sin^{3}\beta +({\overset{–}{Q}}_{36}-{\overset{–}{Q}}_{16}+{\overset{–}{Q}}_{45})\sin\beta \cos^{2}\beta ,\\ K_{55}=({\overset{–}{Q}}_{11}-2{\overset{–}{Q}}_{13}+{\overset{–}{Q}}_{33}-2{\overset{–}{Q}}_{55})\sin^{2}\beta \cos^{2}\beta +{\overset{–}{Q}}_{55}(\sin^{4}\beta +\cos^{4}\beta ),\\ K_{56}={\overset{–}{Q}}_{45}\cos^{3}\beta +({\overset{–}{Q}}_{16}-{\overset{–}{Q}}_{36}-{\overset{–}{Q}}_{45})\sin^{2}\beta \cos\beta ,\\ K_{66}={\overset{–}{Q}}_{44}\cos^{2}\beta +{\overset{–}{Q}}_{66}\sin^{2}\beta ,\;\;\;\;\;K_{ij}=K_{ji},\;i,j=1,\ldots ,6, \end{array}$$

(A2) $$\begin{array}{l} K_{11}^{\psi }=({\overset{–}{Q}}_{31}^{\psi }+{\overset{–}{Q}}_{13}^{\psi }+4{\overset{–}{Q}}_{55}^{\psi })\sin^{2}\beta \cos^{2}\beta +{\overset{–}{Q}}_{11}^{\psi }\sin^{4}\beta +{\overset{–}{Q}}_{33}^{\psi }\cos^{4}\beta ,\\ K_{12}^{\psi }={\overset{–}{Q}}_{12}^{\psi }\sin^{2}\beta +{\overset{–}{Q}}_{23}^{\psi }\cos^{2}\beta ,\\ K_{13}^{\psi }=({\overset{–}{Q}}_{11}^{\psi }+{\overset{–}{Q}}_{33}^{\psi }-4{\overset{–}{Q}}_{55}^{\psi })\sin^{2}\beta \cos^{2}\beta +{\overset{–}{Q}}_{13}^{\psi }\sin^{4}\beta +{\overset{–}{Q}}_{31}^{\psi }\cos^{4}\beta ,\\ K_{14}^{\psi }=({\overset{–}{Q}}_{16}^{\psi }-2{\overset{–}{Q}}_{45}^{\psi })\sin^{2}\beta \cos\beta +{\overset{–}{Q}}_{36}^{\psi }\cos^{3}\beta ,\\ K_{15}^{\psi }=({\overset{–}{Q}}_{13}^{\psi }-{\overset{–}{Q}}_{11}^{\psi }+2{\overset{–}{Q}}_{55}^{\psi })\sin^{3}\beta \cos\beta +({\overset{–}{Q}}_{33}^{\psi }-{\overset{–}{Q}}_{31}^{\psi }-2{\overset{–}{Q}}_{55}^{\psi })\sin\beta \cos^{3}\beta ,\\ K_{16}^{\psi }=-{\overset{–}{Q}}_{16}^{\psi }\sin^{3}\beta -(2{\overset{–}{Q}}_{45}^{\psi }+{\overset{–}{Q}}_{36}^{\psi })\sin\beta \cos^{2}\beta ,\\ K_{21}^{\psi }={\overset{–}{Q}}_{21}^{\psi }\sin^{2}\beta +{\overset{–}{Q}}_{23}^{\psi }\cos^{2}\beta ,\;\;\;\;K_{22}^{\psi }={\overset{–}{Q}}_{22}^{\psi },\\ K_{23}^{\psi }={\overset{–}{Q}}_{21}^{\psi }\cos^{2}\beta +{\overset{–}{Q}}_{23}^{\psi }\sin^{2}\beta ,\;\;\;\;K_{24}^{\psi }={\overset{–}{Q}}_{26}^{\psi }\cos\beta ,\\ K_{25}^{\psi }=({\overset{–}{Q}}_{23}^{\psi }-{\overset{–}{Q}}_{21}^{\psi })\sin\beta \cos\beta ,\;\;\;\;K_{26}^{\psi }=-{\overset{–}{Q}}_{26}^{\psi }\sin\beta ,\\ K_{31}^{\psi }=({\overset{–}{Q}}_{11}^{\psi }+{\overset{–}{Q}}_{33}^{\psi }-4{\overset{–}{Q}}_{55}^{\psi })\sin^{2}\beta \cos^{2}\beta +{\overset{–}{Q}}_{31}^{\psi }\sin^{4}\beta +{\overset{–}{Q}}_{13}^{\psi }\cos^{4}\beta ,\\ K_{32}^{\psi }={\overset{–}{Q}}_{12}^{\psi }\cos^{2}\beta +{\overset{–}{Q}}_{23}^{\psi }\sin^{2}\beta ,\\ K_{33}^{\psi }=({\overset{–}{Q}}_{31}^{\psi }+{\overset{–}{Q}}_{13}^{\psi }+4{\overset{–}{Q}}_{55}^{\psi })\sin^{2}\beta \cos^{2}\beta +{\overset{–}{Q}}_{33}^{\psi }\sin^{4}\beta +{\overset{–}{Q}}_{11}^{\psi }\cos^{4}\beta ,\\ K_{34}^{\psi }=(2{\overset{–}{Q}}_{45}^{\psi }+{\overset{–}{Q}}_{36}^{\psi })\sin^{2}\beta \cos\beta +{\overset{–}{Q}}_{16}^{\psi }\cos^{3}\beta ,\\ K_{35}^{\psi }=({\overset{–}{Q}}_{13}^{\psi }-{\overset{–}{Q}}_{11}^{\psi }+2{\overset{–}{Q}}_{55}^{\psi })\sin\beta \cos^{3}\beta +({\overset{–}{Q}}_{33}^{\psi }-{\overset{–}{Q}}_{31}^{\psi }-2{\overset{–}{Q}}_{55}^{\psi })\sin^{3}\beta \cos\beta ,\\ K_{36}^{\psi }=(2{\overset{–}{Q}}_{45}^{\psi }-{\overset{–}{Q}}_{16}^{\psi })\sin\beta \cos^{2}\beta +{\overset{–}{Q}}_{36}^{\psi }\sin^{3}\beta ,\\ K_{41}^{\psi }=({\overset{–}{Q}}_{61}^{\psi }-2{\overset{–}{Q}}_{45}^{\psi })\sin^{2}\beta \cos\beta +{\overset{–}{Q}}_{63}^{\psi }\cos^{3}\beta ,\;\;\;\;K_{42}^{\psi }={\overset{–}{Q}}_{62}^{\psi }\cos\beta ,\\ K_{43}^{\psi }=(2{\overset{–}{Q}}_{45}^{\psi }+{\overset{–}{Q}}_{63}^{\psi })\sin^{2}\beta \cos\beta +{\overset{–}{Q}}_{61}^{\psi }\cos^{3}\beta ,\\ K_{44}^{\psi }={\overset{–}{Q}}_{44}^{\psi }\sin^{2}\beta +{\overset{–}{Q}}_{66}^{\psi }\cos^{2}\beta ,\\ K_{45}^{\psi }=({\overset{–}{Q}}_{63}^{\psi }-{\overset{–}{Q}}_{61}^{\psi }+{\overset{–}{Q}}_{45}^{\psi })\sin\beta \cos^{2}\beta -{\overset{–}{Q}}_{45}^{\psi }\sin^{3}\beta ,\\ K_{46}^{\psi }=({\overset{–}{Q}}_{44}^{\psi }-{\overset{–}{Q}}_{66}^{\psi })\sin\beta \cos\beta ,\\ K_{51}^{\psi }=({\overset{–}{Q}}_{31}^{\psi }-{\overset{–}{Q}}_{11}^{\psi }+2{\overset{–}{Q}}_{55}^{\psi })\sin^{3}\beta \cos\beta +({\overset{–}{Q}}_{33}^{\psi }-{\overset{–}{Q}}_{13}^{\psi }-2{\overset{–}{Q}}_{55}^{\psi })\sin\beta \cos^{3}\beta ,\\ K_{52}^{\psi }=({\overset{–}{Q}}_{23}^{\psi }-{\overset{–}{Q}}_{12}^{\psi })\sin\beta \cos\beta ,\\ K_{53}^{\psi }=({\overset{–}{Q}}_{31}^{\psi }-{\overset{–}{Q}}_{11}^{\psi }+2{\overset{–}{Q}}_{55}^{\psi })\sin\beta \cos^{3}\beta +({\overset{–}{Q}}_{33}^{\psi }-{\overset{–}{Q}}_{13}^{\psi }-2{\overset{–}{Q}}_{55}^{\psi })\sin^{3}\beta \cos\beta ,\\ K_{54}^{\psi }=({\overset{–}{Q}}_{36}^{\psi }-{\overset{–}{Q}}_{16}^{\psi }+{\overset{–}{Q}}_{45}^{\psi })\sin\beta \cos^{2}\beta -{\overset{–}{Q}}_{45}^{\psi }\sin^{3}\beta ,\\ K_{55}^{\psi }=({\overset{–}{Q}}_{11}^{\psi }+{\overset{–}{Q}}_{33}^{\psi }-{\overset{–}{Q}}_{31}^{\psi }-{\overset{–}{Q}}_{13}^{\psi }-2{\overset{–}{Q}}_{55}^{\psi })\sin^{2}\beta \cos^{2}\beta +{\overset{–}{Q}}_{55}^{\psi }(\sin^{4}\beta +\cos^{4}\beta ),\\ K_{56}^{\psi }={\overset{–}{Q}}_{45}^{\psi }\cos^{3}\beta +({\overset{–}{Q}}_{16}^{\psi }-{\overset{–}{Q}}_{36}^{\psi }-{\overset{–}{Q}}_{45}^{\psi })\sin^{2}\beta \cos\beta ,\\ K_{61}^{\psi }=-{\overset{–}{Q}}_{61}^{\psi }\sin^{3}\beta -({\overset{–}{Q}}_{63}^{\psi }+2{\overset{–}{Q}}_{45}^{\psi })\sin\beta \cos^{2}\beta ,\;\;\;\;K_{62}^{\psi }=-{\overset{–}{Q}}_{62}^{\psi }\sin\beta ,\\ K_{63}^{\psi }=-{\overset{–}{Q}}_{63}^{\psi }\sin^{3}\beta +(2{\overset{–}{Q}}_{45}^{\psi }-{\overset{–}{Q}}_{61}^{\psi })\sin\beta \cos^{2}\beta ,\;\;\;\;K_{64}^{\psi }=({\overset{–}{Q}}_{44}^{\psi }-{\overset{–}{Q}}_{66}^{\psi })\sin\beta \cos\beta ,\\ K_{65}^{\psi }=({\overset{–}{Q}}_{61}^{\psi }-{\overset{–}{Q}}_{63}^{\psi }-{\overset{–}{Q}}_{45}^{\psi })\sin^{2}\beta \cos\beta +{\overset{–}{Q}}_{45}^{\psi }\cos^{3}\beta ,\;\;\;\;K_{66}^{\psi }={\overset{–}{Q}}_{44}^{\psi }\cos^{2}\beta +{\overset{–}{Q}}_{66}^{\psi }\sin^{2}\beta , \end{array}$$

where

(A3) $$\begin{array}{l} {\bar{Q}}_{11}=Q_{11}\cos^{4}\alpha +2(Q_{12}+2Q_{66})\sin^{2}\alpha \cos^{2}\alpha +Q_{22}\sin^{4}\alpha ,\;\;\;{\bar{Q}}_{13}=Q_{13}\cos^{2}\alpha +Q_{23}\sin^{2}\alpha ,\\ {\bar{Q}}_{12}=(Q_{11}+Q_{22}-4Q_{66})\sin^{2}\alpha \cos^{2}\alpha +Q_{12}(\sin^{4}\alpha +\cos^{4}\alpha ),\;\;\;{\bar{Q}}_{23}=Q_{13}\sin^{2}\alpha +Q_{23}\cos^{2}\alpha ,\\ {\bar{Q}}_{16}=(Q_{11}-Q_{12}-2Q_{66})\sin\alpha \cos^{3}\alpha +(Q_{12}-Q_{22}+2Q_{66})\sin^{3}\alpha \cos\alpha ,\\ {\bar{Q}}_{22}=Q_{11}\sin^{4}\alpha +2(Q_{12}+2Q_{66})\sin^{2}\alpha \cos^{2}\alpha +Q_{22}\cos^{4}\alpha ,\;\;\;{\bar{Q}}_{36}=(Q_{13}-Q_{23})\sin\alpha \cos\alpha ,\\ {\bar{Q}}_{26}=(Q_{11}-Q_{12}-2Q_{66})\sin^{3}\alpha \cos\alpha +(Q_{12}-Q_{22}+2Q_{66})\sin\alpha \cos^{3}\alpha ,\\ {\bar{Q}}_{33}=Q_{33},\;\;\;\;\;{\bar{Q}}_{44}=Q_{44}\cos^{2}\alpha +Q_{55}\sin^{2}\alpha ,\;\;\;\;\;{\bar{Q}}_{45}=(Q_{55}-Q_{44})\sin\alpha \cos\alpha ,\\ {\bar{Q}}_{66}=(Q_{11}+Q_{22}-2Q_{12}-2Q_{66})\sin^{2}\alpha \cos^{2}\alpha +Q_{66}(\sin^{4}\alpha +\cos^{4}\alpha ),\\ {\bar{Q}}_{55}=Q_{44}\sin^{2}\alpha +Q_{55}\cos^{2}\alpha ,\;\;\;\;{\bar{Q}}_{i,j}={\bar{Q}}_{j,i}\;,i,j=1,\ldots ,6, \end{array}$$

(A4) $$\begin{array}{l} {\bar{Q}}_{11}^{\psi }=0.5\pi [(Q_{12}\psi_{1}+Q_{12}\psi_{2}+4Q_{66}\psi_{12})\sin^{2}\alpha \cos^{2}\alpha +Q_{22}\psi_{2}\sin^{4}\alpha +Q_{11}\psi_{1}\cos^{4}\alpha ],\\ {\bar{Q}}_{12}^{\psi }=0.5\pi [(Q_{11}\psi_{1}+Q_{22}\psi_{2}-4Q_{66}\psi_{12})\sin^{2}\alpha \cos^{2}\alpha +Q_{12}\psi_{1}\sin^{4}\alpha +Q_{12}\psi_{2}\cos^{4}\alpha ],\\ {\bar{Q}}_{13}^{\psi }=0.5\pi (Q_{13}\psi_{3}\cos^{2}\alpha +Q_{23}\psi_{3}\sin^{2}\alpha ),\;\;\;\;{\bar{Q}}_{23}^{\psi }=0.5\pi (Q_{13}\psi_{3}\sin^{2}\alpha +Q_{23}\psi_{3}\cos^{2}\alpha ),\\ {\bar{Q}}_{16}^{\psi }=0.5\pi [(Q_{11}\psi_{1}-Q_{12}\psi_{2}-2Q_{66}\psi_{12})\sin\alpha \cos^{3}\alpha +(Q_{12}\psi_{1}-Q_{22}\psi_{2}+2Q_{66}\psi_{12})\sin^{3}\alpha \cos\alpha ],\\ {\bar{Q}}_{21}^{\psi }=0.5\pi [(Q_{11}\psi_{1}+Q_{22}\psi_{2}-4Q_{66}\psi_{12})\sin^{2}\alpha \cos^{2}\alpha +Q_{12}\psi_{2}\sin^{4}\alpha +Q_{12}\psi_{1}\cos^{4}\alpha ],\\ {\bar{Q}}_{22}^{\psi }=0.5\pi [(Q_{12}\psi_{1}+Q_{12}\psi_{2}+4Q_{66}\psi_{12})\sin^{2}\alpha \cos^{2}\alpha +Q_{11}\psi_{1}\sin^{4}\alpha +Q_{22}\psi_{2}\cos^{4}\alpha ],\\ {\bar{Q}}_{26}^{\psi }=0.5\pi [(Q_{11}\psi_{1}-Q_{12}\psi_{2}-2Q_{66}\psi_{12})\sin^{3}\alpha \cos\alpha +(Q_{12}\psi_{1}-Q_{22}\psi_{2}+2Q_{66}\psi_{12})\sin\alpha \cos^{3}\alpha ],\\ {\bar{Q}}_{31}^{\psi }=0.5\pi (Q_{13}\psi_{1}\cos^{2}\alpha +Q_{23}\psi_{2}\sin^{2}\alpha ),\;\;\;\;{\bar{Q}}_{32}^{\psi }=0.5\pi (Q_{13}\psi_{1}\sin^{2}\alpha +Q_{23}\psi_{2}\cos^{2}\alpha ),\\ {\bar{Q}}_{33}^{\psi }=0.5\pi Q_{33}\psi_{3},\;\;\;\;\;\;\;\;\;\;\;\;\;{\bar{Q}}_{36}^{\psi }=0.5\pi (Q_{13}\psi_{1}-Q_{23}\psi_{2})\sin\alpha \cos\alpha ,\\ {\bar{Q}}_{44}^{\psi }=0.5\pi (Q_{44}\psi_{23}\cos^{2}\alpha +Q_{55}\psi_{13}\sin^{2}\alpha ),\;\;\;\;{\bar{Q}}_{45}^{\psi }=0.5\pi (Q_{55}\psi_{13}-Q_{44}\psi_{23})\sin\alpha \cos\alpha ,\\ {\bar{Q}}_{54}^{\psi }={\bar{Q}}_{45}^{\psi },\;\;\;\;\;\;\;\;\;\;\;\;\;\;\;{\bar{Q}}_{55}^{\psi }=0.5\pi (Q_{55}\psi_{13}\cos^{2}\alpha +Q_{44}\psi_{23}\sin^{2}\alpha ),\\ {\bar{Q}}_{61}^{\psi }=0.5\pi [(Q_{11}\psi_{1}-Q_{12}\psi_{1}-2Q_{66}\psi_{12})\sin\alpha \cos^{3}\alpha +(Q_{12}\psi_{2}-Q_{22}\psi_{2}+2Q_{66}\psi_{12})\sin^{3}\alpha \cos\alpha ],\\ {\bar{Q}}_{62}^{\psi }=0.5\pi [(Q_{11}\psi_{1}-Q_{12}\psi_{1}-2Q_{66}\psi_{12})\sin^{3}\alpha \cos\alpha +(Q_{12}\psi_{2}-Q_{22}\psi_{2}+2Q_{66}\psi_{12})\sin\alpha \cos^{3}\alpha ],\\ {\bar{Q}}_{63}^{\psi }=0.5\pi (Q_{13}-Q_{23})\psi_{3}\sin\alpha \cos\alpha ,\\ {\bar{Q}}_{66}^{\psi }=0.5\pi [(Q_{11}\psi_{1}-Q_{12}\psi_{1}-Q_{12}\psi_{2}+Q_{22}\psi_{2})\sin^{2}\alpha \cos^{2}\alpha +Q_{66}\psi_{12}{\left(\sin^{2}\alpha -\cos^{2}\alpha \right)}^{2}]. \end{array}$$

Substituting the above transformation into the stress–strain equation, the following can be obtained:

(A5) $$\left\{\sigma \right\}=\{\begin{array}{l} \sigma_{xx}={\overset{–}{K}}_{11}\varepsilon_{xx}+{\overset{–}{K}}_{11}^{\psi }{\dot{\varepsilon }}_{xx}\\ \tau_{xz}=k_{s}{\overset{–}{K}}_{12}\gamma_{xz}+k_{s}{\overset{–}{K}}_{12}^{\psi }{\dot{\gamma }}_{xz}\\ \tau_{xy}=k_{s}{\overset{–}{K}}_{13}\gamma_{xy}+k_{s}{\overset{–}{K}}_{13}^{\psi }{\dot{\gamma }}_{xy} \end{array}$$

where $k_{s}$ is the shear correction factor. For the Euler–Bernoulli beam theory without considering the transverse shear deformation, $k_{s}$ = 0 was taken. ${\overset{–}{K}}_{ij}$ and ${\overset{–}{K}}_{ij}^{\psi }$ are related to the ply angle of the fiber and the elastic constant of the main direction of the material, which can be expressed as [16]

(A6) $$\begin{array}{l} {\overset{–}{K}}_{11}=K_{33}+\left(K_{13}\Delta_{K}^{13}+K_{23}\Delta_{K}^{23}+K_{36}\Delta_{K}^{36}\right)/\Delta_{K},{\overset{–}{K}}_{12}^{\psi }=K_{34}^{\psi }+\left(K_{14}^{\psi }\Delta_{K}^{14}{}^{\psi }+K_{24}^{\psi }\Delta_{K}^{24}{}^{\psi }+K_{46}^{\psi }\Delta_{K}^{46}{}^{\psi }\right)/\Delta_{K}^{\psi },\\ {\overset{–}{K}}_{13}=K_{35}+\left(K_{15}\Delta_{K}^{15}+K_{25}\Delta_{K}^{25}+K_{56}\Delta_{K}^{56}\right)/\Delta_{K},{\overset{–}{K}}_{11}^{\psi }=K_{33}^{\psi }+\left(K_{13}^{\psi }\Delta_{K}^{13}{}^{\psi }+K_{23}^{\psi }\Delta_{K}^{23}{}^{\psi }+K_{36}^{\psi }\Delta_{K}^{36}{}^{\psi }\right)/\Delta_{K}^{\psi },\\ {\overset{–}{K}}_{12}=K_{34}+\left(K_{14}\Delta_{K}^{14}+K_{24}\Delta_{K}^{24}+K_{46}\Delta_{K}^{46}\right)/\Delta_{K},{\overset{–}{K}}_{13}^{\psi }=K_{35}^{\psi }+\left(K_{15}^{\psi }\Delta_{K}^{15}{}^{\psi }+K_{25}^{\psi }\Delta_{K}^{25}{}^{\psi }+K_{56}^{\psi }\Delta_{K}^{56}{}^{\psi }\right)/\Delta_{K}^{\psi }. \end{array}$$

where

(A7) $$\begin{array}{l} {K_{i6}}^{\prime }=K_{i6}+K_{i4}\tan\beta ,\;\;\;\;\;\;\;{K_{6i}^{\psi }}^{\prime }=K_{6i}^{\psi }+K_{4i}^{\psi }\tan\beta ,\;\;\;\;\;\;\;\;\;\;\;\;i=1...6;\\ \Delta_{K}=\left|\begin{array}{ccc} K_{11} & K_{12} & K_{16}\\ K_{12} & K_{22} & K_{26}\\ {K_{16}}^{\prime } & {K_{26}}^{\prime } & {K_{66}}^{\prime } \end{array}\right|,\;\Delta_{K}^{\psi }=\left|\begin{array}{ccc} K_{11}^{\psi } & K_{12}^{\psi } & K_{16}^{\psi }\\ K_{21}^{\psi } & K_{22}^{\psi } & K_{26}^{\psi }\\ {K_{61}^{\psi }}^{\prime } & {K_{62}^{\psi }}^{\prime } & {K_{66}^{\psi }}^{\prime } \end{array}\right|,\;\Delta_{K}^{1i}=\left|\begin{array}{ccc} -K_{1i} & K_{12} & K_{16}\\ -K_{2i} & K_{22} & K_{26}\\ -{K_{i6}}^{\prime } & {K_{26}}^{\prime } & {K_{66}}^{\prime } \end{array}\right|,\\ \Delta_{K}^{2i}=\left|\begin{array}{ccc} K_{11} & -K_{1i} & K_{16}\\ K_{12} & -K_{2i} & K_{26}\\ {K_{16}}^{\prime } & -{K_{i6}}^{\prime } & {K_{66}}^{\prime } \end{array}\right|,\;\Delta_{K}^{i6}=\left|\begin{array}{ccc} K_{11} & K_{12} & -K_{1i}\\ K_{12} & K_{22} & -K_{2i}\\ {K_{16}}^{\prime } & {K_{26}}^{\prime } & -{K_{i6}}^{\prime } \end{array}\right|,\;\Delta_{K}^{1i}{}^{\psi }=\left|\begin{array}{ccc} -K_{1i}^{\psi } & K_{12}^{\psi } & K_{16}^{\psi }\\ -K_{2i}^{\psi } & K_{22}^{\psi } & K_{26}^{\psi }\\ -{K_{6i}^{\psi }}^{\prime } & {K_{62}^{\psi }}^{\prime } & {K_{66}^{\psi }}^{\prime } \end{array}\right|,\\ \Delta_{K}^{2i}{}^{\psi }=\left|\begin{array}{ccc} K_{11}^{\psi } & -K_{1i}^{\psi } & K_{16}^{\psi }\\ K_{21}^{\psi } & -K_{2i}^{\psi } & K_{26}^{\psi }\\ {K_{61}^{\psi }}^{\prime } & -{K_{6i}^{\psi }}^{\prime } & {K_{66}^{\psi }}^{\prime } \end{array}\right|,\;\Delta_{K}^{i6}{}^{\psi }=\left|\begin{array}{ccc} K_{11}^{\psi } & K_{12}^{\psi } & -K_{1i}^{\psi }\\ K_{21}^{\psi } & K_{22}^{\psi } & -K_{2i}^{\psi }\\ {K_{61}^{\psi }}^{\prime } & {K_{62}^{\psi }}^{\prime } & -{K_{6i}^{\psi }}^{\prime } \end{array}\right|,\;\;\;\;\;i=3,4,5. \end{array}$$

## References

[1] Hao C., Zhen D., Bing Z.Z., Shuai C. (2021). Design of a new type of deep hole boring device. J. Phys. Conf. Ser..

[2] Sino R., Baranger T.N., Chatelet E., Jacquet G. (2008). Dynamic analysis of a rotating composite shaft. Compos. Sci. Technol..

[3] Moetakef-Imani B., Yussefian N. (2009). Dynamic simulation of boring process. Int. J. Mach. Tool Manuf..

[4] Merritt H.E. (1965). Theory of self-excited machine-tool chatter: Contribution to machine-tool chatter research—1. J. Eng. Ind..

[5] Insperger T., Stepan G. (2007). Act-and-wait control concept for discrete-time systems with feedback delay. IET Control. Theory Appl..

[6] Altintas Y., Weck M. (2004). Chatter Stability of Metal Cutting and Grinding. CIRP Ann. Manuf. Technol..

[7] Solis E., Peres C.R., Jiménez J.E., Alique J.R., Monje J.C. (2004). A new analytical–experimental method for the identification of stability lobes in high-speed milling. Int. J. Mach. Tool Manuf..

[8] Li M., Zhang G., Yu H. (2013). Complete discretization scheme for milling stability prediction. Nonlinear Dyn..

[9] Zhou X., Yu D., Shao X., Zhang S., Wang S. (2016). Research and applications of viscoelastic vibration damping materials: A review. Compos. Struct..

[10] Treviso A., van Genechten B., Mundo D., Tournour M. (2015). Damping in composite materials: Properties and models. Compos. Part B.

[11] Mendonça W.R.D.P., de Medeiros E.C., Pereira A.L.R., Mathias M.H. (2017). The dynamic analysis of rotors mounted on composite shafts with internal damping. Compos. Struct..

[12] Montagnier O., Hochard C. (2014). Dynamics of a supercritical composite shaft mounted on viscoelastic supports. J. Sound Vib..

[13] Denghui L., Hongrui C., Xuefeng C. (2022). Active control of milling chatter considering the coupling effect of spindle-tool and workpiece systems. Mech. Syst. Signal Process..

[14] Molnar G.T., Insperger T., Hogan J.S., Stepan G. (2016). Estimation of the Bistable Zone for Machining Operations for the Case of a Distributed Cutting-Force Model. J. Comput. Nonlinear Dyn..

[15] Qinliang L., Bo W., Bin Z., Bangchun W. (2013). Research on the Chatter Stability of Machine System Taking the Nonlinear Hysteretic Force into Consi. Chin. J. Mech. Eng..

[16] Kim W. (1999). Vibration of a Rotating Tapered Composite Shaft and Applications to High Speed Cutting.

[17] Tian J., Hutton S.G. (2001). Chatter instability in milling systems with flexible rotating spindles—A new theoretical approach. J. Manuf. Sci. Eng..

[18] Jingmin M., Yongsheng R. (2018). Free vibration and chatter stability of a rotating thin-walled composite bar. Adv. Mech. Eng.

[19] Jingmin M., Jianfeng X., Yongsheng R. (2020). Analysis on free vibration and stability of rotating composite milling bar with large aspect ratio. Appl. Sci..

[20] Kapoor S.G., Zhang G.M., Bahney L.L. (1984). Stability analysis of the boring process system. Manuf. Eng. Trans..

[21] Subramani G., Kapoor S.G., Devor R.E. (1993). A model for the prediction of bore cylindricity during machining. J. Eng. Ind..

[22] Kim W., Argento A., Scott R.A. (2001). Forced vibration and dynamic stability of a rotating tapered composite timoshenko shaft: Bending motions in end-milling operations. JSV.

[23] Smith S., Tlusty J. (1991). An overview of modeling and simulation of the milling process. J. Eng. Ind..

